# ProCAPTCHA: A profile-based CAPTCHA for personal password authentication

**DOI:** 10.1371/journal.pone.0311197

**Published:** 2024-12-05

**Authors:** Nilobon Nanglae, Pattarasinee Bhattarakosol

**Affiliations:** Department of Mathematics and Computer Science, Faculty of Science, Chulalongkorn University, Bangkok, Thailand; National University of Sciences and Technology, UNITED KINGDOM OF GREAT BRITAIN AND NORTHERN IRELAND

## Abstract

CAPTCHA was introduced decades ago to distinguish between humans and bots. However, solving CAPTCHA has been a challenging issue for intruders. Various techniques, such as 3rd-party attacks, have been invented to break CAPTCHA. This research proposes ProCAPTCHA, a CAPTCHA system individually generated for each user by merging biometrics and user profiles. ProCAPTCHA leverages keystroke dynamics and personal information to create unique CAPTCHAs that are difficult for intruders to solve. ProCAPTCHA’s algorithm generates CAPTCHA based on the user’s profile data, ensuring randomness and uniqueness for each login. Performance evaluation shows that ProCAPTCHA can identify legitimate users with 100% accuracy, while only 60% of intruders are misclassified as true users. Bots face significant delays, often failing due to system time limits. Nonetheless, the bot’s attack must spend a very long time solving which, in real life, could be interrupted by the time limit of the system. Therefore, all bots cannot gain access as required.

## 1. Introduction

The internet has become an essential part of daily life, with people using many online services daily. Unfortunately, this widespread access has also made the internet a prime target for malicious activities; one significant attack is automated bot attacks. These attacks can be carried out at a massive scale, with bots capable of creating fake accounts, submitting spam comments, or launching DDoS attacks that can bring down entire websites [1–3]. However, CAPTCHA (Completely Automated Public Turing Test to Tell Computers and Humans Apart) technology has been developed to distinguish bots from real human beings, and it plays an essential role in mitigating the impact of automated attacks on online services [4]. By providing a barrier that is difficult for bots to overcome, CAPTCHA helps ensure the security and reliability of online services, protecting both the service provider and the user. Therefore, the development and widespread adoption of CAPTCHA technology has been crucial in maintaining a safe and secure online environment and protecting against bot attacks.

While CAPTCHA technology has effectively prevented automated bot attacks, some problems with it that can compromise its security. One problem is that bots are getting better at getting around CAPTCHAs, which makes it easier for them to get around security measures. To address this issue, some CAPTCHA providers have developed more advanced forms of CAPTCHA [5], such as image-based CAPTCHAs [6] and video-based CAPTCHAs [7]. These forms of CAPTCHA are more difficult for bots to solve because they require more complex image recognition or video analysis algorithms. Therefore, some CAPTCHA providers have developed adaptive CAPTCHA systems that adjust the difficulty level of the challenge based on the user’s behavior. For example, NuCAPTCHA has an "Adaptive Security" feature that adjusts the difficulty level of the CAPTCHA challenge based on the user’s behavior. If the user consistently enters the correct characters, the system may increase the complexity of the challenge to make it more difficult for bots to pass [8]. This helps ensure that bots cannot adapt to the CAPTCHA system and remain effective in malicious protection. Although CAPTCHA technology has effectively prevented automated bot attacks, the increasing sophistication of bots and the emergence of new types of attacks, such as third-party attacks, pose a challenge for CAPTCHA providers. As a result, ongoing innovation and development of advanced CAPTCHAs are necessary to ensure the security and reliability of online services. It is crucial to stay ahead of these evolving threats to maintain the integrity of online platforms and protect users from potential harm.

Third-party attacks are a type of attack where malicious actors attempt to bypass CAPTCHA systems by outsourcing the task to a third party. These third-party services typically employ low-cost human labor from countries with lower wages, such as India or Bangladesh, to solve CAPTCHAs on behalf of the attackers [9]. This allows the attackers to bypass security measures and access online services and resources [10]. Text-based CAPTCHAs are particularly vulnerable to third-party attacks because they are the most widely used type of CAPTCHA, due to their simplicity and ease of implementation [11]. As a result, attackers can efficiently train their human workers to recognize and solve these types of CAPTCHAs, making them less effective at preventing unauthorized access.

To address this issue, many online services have begun implementing MFA (Multi-factor authentication) as an additional layer of security. MFA requires users to provide multiple forms of authentication, such as a password and a unique code sent to their mobile device, to gain access to an account or service. This makes it more difficult for attackers to gain access, even if they bypass the CAPTCHA system. MFA, which adds another layer of security on top of the CAPTCHA, is another way to solve the problem. MFA requires users to provide two or more forms of authentication, such as a password and a fingerprint scan or a one-time code sent to their mobile device. This makes it much more difficult for bots and third-party attacks to bypass the security measures, even if they can solve the CAPTCHA.

CAPTCHA has evolved over the years to include different variations, primarity text-based CAPTCHA. Humans and robots have differentiated recognition skills when presented with CAPTCHAs of varying stripes, distorted letters, or contents. Most of the time, humans can classify complicated CAPTCHAs better than bots. Nevertheless, users need help identifying the proper text or characters of CAPTCHAs when the CAPTCHAs are composed of high distortion of letters and stripes.

## 2. Related work

According to Google, CAPTCHA is used on over 4 million websites worldwide [12], and it had become a standard tool for preventing automated bot submissions on online forms. Recently, text-based CAPTCHA has become popular and widely recognized. However, this type of CAPTCHA can be attacked by two primary approaches. The first approach uses OCR (Optical Character Recognition) technology to defeat the traditional printed format of the text-based CAPTCHA [13]. The second approach is using CAPTCHA farms, where third parties hire humans to solve CAPTCHAs that are captured from the target websites.

### 2.1 OCR attacks and solutions

Since traditional text-based CAPTCHAs are easy to solve, various implementations have been proposed to protect against breaking algorithms. In the early stages of altering text-based CAPTCHAs 2003, Von Ahn et al. proposed using graphical schemes in CAPTCHAs, representing text-based CAPTCHAs with 2D or 3D letter shapes. Despite their innovation, these CAPTCHAs were still vulnerable to sophisticated OCR attacks and could be challenging for users with visual impairments [4]. In 2004, Rusu and Govindaraju introduced handwritten CAPTCHAs to exploit the difference in reading abilities between humans and machines. However, this method was only sometimes applicable, particularly for non-English speakers and individuals with visual impairments. The complexity of recognizing diverse handwriting styles further exacerbated these challenges, making the CAPTCHA less user-friendly for a broad audience [14]. Then [15], introduced the idea of a clickable CAPTCHA that combines various textual CAPTCHAs into a clickable grid of CAPTCHAs (e.g., a 3-by-4 grid). Thus, users need to click on the required grids; those are English words. Although the clickable grid of CAPTCHAs is efficient, it is unsuitable for non-English speakers because they may be unable to read or understand the words in the grids. In 2016, NuCAPTCHA employed video-based CAPTCHAs to enhance security through motion analysis. While more secure, this method was resource-intensive and could cause delays for users on slower internet connections [16]. To address these issues, Kim and Choi in (2019) introduced animated CAPTCHAs that required identifying moving objects. This approach made it more complicated for computers to comprehend the challenge but was frustrating for users, especially those with visual or cognitive disabilities [17]. To eliminate such problems, the implementation of 2D media, 3D media, or animated media have been proposed [16–18]. This technique represents text-based CAPTCHA with graphical scheme as 2D or 3D letter shapes.

Although the 2D text-based CAPTCHA and the clickable grid of CAPTCHAs have been presented, these techniques are still vulnerable to intrusion. Therefore, 3D CAPTCHAs have been introduced to make characters more realistic and challenging for computers to understand. Furthermore, an animated CAPTCHA that uses motion and animation makes it harder for computers to comprehend the challenge. Thus, these types of CAPTCHAs– 3D and animated CAPTCHAs–might require the user to identify moving objects or follow a specific pattern in a series of moving characters. The main problem with all CAPTCHA types is that they can sometimes be difficult for humans to solve, especially those with visual impairments or cognitive disabilities. Some users may also find them frustrating or time-consuming, negatively impacting the user experience.

Even though the OCR attacks have been studied for years, this research will focus only the CAPTCHA farms attacks that is described in the following section.

### 2.2 CAPTCHA farms attacks and solution

In recent years, one of the main problems with text-based CAPTCHA attacks has been CAPTCHA farms, where a group of third parties is hired to break the CAPTCHAs in a certain period [19]. The third-party services bypass or solve CAPTCHA challenges by outsourcing the task of solving the challenge to a third-party provider. These services typically employ human workers who are paid a small compensation to break CAPTCHA challenges. Then, the answers are sent to the intruders who want to pass the security detection. CAPTCHA farms work by using a distributed workforce of human solvers who are trained to solve CAPTCHA challenges quickly and efficiently. These solvers are typically located in developing countries with low labor income and they may work on multiple CAPTCHA challenges simultaneously. Using third-party services to solve CAPTCHA challenges poses several potential security risks to websites and users.

Over the years, several creative CAPTCHA solutions have emerged to address the problem that CAPTCHA farms pose. Early CAPTCHAs, like audio and puzzles, were initially effective but were eventually bypassed by advanced bots. This led to the development of more robust security measures. In 2018, Frank et al. suggested that users make three-dimensional shapes or words while holding the device, which the gyroscope could identify. However, this approach relied heavily on the availability and accuracy of gyroscope sensors, limiting its applicability to devices without such sensors [20]. CAPTCHA methods that rely on behavioral biometrics, such as mouse dynamics, swipe dynamics, and eye movement, may be less accessible to users with physical impairments. Users with motor disabilities may struggle with activities that call for precise mouse movements or eye tracking, while users without sight may struggle with tasks that call for visual clues.

Furthermore, these methods may need to be more effective against sophisticated bots programmed to appear human. In 2014, Google’s introduction of "No CAPTCHA reCAPTCHA" represented a significant milestone in CAPTCHA technology, which aimed to distinguish humans from bots by asking users to check a box, utilizing behavioral analysis for verification simply. Despite its capabilities, it still faced challenges with more advanced bots that could effectively mimic human behavior [21, 22]. The following year, in 2015, Guerar et al. introduced CAPPCHA, a groundbreaking concept for mobile device CAPTCHAs. CAPPCHA introduced a novel requirement for users to tilt their devices at specific angles, eliminating the need for complex actions or unpredictable challenges thanks to specialized hardware sensors known for their heightened sensitivity. However, this method depend on the availability and accuracy of these sensors, limiting its applicability [23]. In 2016, Hupperich et al. unveiled Sensor CAPTCHA [24], which harnessed user motion as a means of human verification. While innovative, this approach was challenging for users with physical impairments and required specific hardware capabilities. The same year saw advancements in CAPTCHAs rooted in behavioral biometrics, including keystroke and mouse dynamics, swipe patterns, and eye movements. However, users with physical impairments may find these methods less accessible, and they may prove ineffective against sophisticated bots programmed to appear human.

Advancements also emerged in CAPTCHAs rooted in behavioral biometrics, encompassing keystroke and mouse dynamics, swipe patterns, and eye movements. In 2017, Sushama Kulkarni and H. S. Fadewar introduced pedometric CAPTCHAs [25], which required users to perform a sequence of typically five or more steps to confirm their human identity. This approach aimed to leverage mobile device acceleration in a problematic way for bots to replicate, especially when users were in motion. However, it could be impractical for users in stationary environments or those with mobility issues. In the same year, Google’s introduction of "Invisible reCAPTCHA," an enhanced iteration of reCAPTCHA V2, marked a pivotal development. This version assessed user behavior to gauge the likelihood of encountering a bot before triggering additional verification steps [26, 27]. A notable example is EYE-CAPTCHA, which used eye movement for verification. This system excluded users with visual impairments and required specialized eye-tracking hardware [28]. Furthermore, in 2018, Mantri et al. introduced an innovative CAPTCHA approach that required users to align their devices with a predetermined on-screen pattern. For example, executing the "submit" command involved tracing the letter "S" while holding the device in a specific manner. However, this method could be cumbersome and dependent on the user’s ability to align their device precisely [29]. Also, CAPTCHAs based on behavioral biometrics, including keystroke and mouse dynamics, swipe patterns, and eye movements, have been used in different ways, such as Gametrics, GEETest, and Netease [30]. Gametrics uses gamified CAPTCHAs to distinguish humans from bots, making challenges enjoyable and engaging. However, sophisticated bots can sometimes mimic these interactions, reducing their effectiveness. GEETest offers slide and click-based CAPTCHAs, leveraging behavioral biometrics for enhanced security. However, advanced machine learning bots can bypass these CAPTCHAs, posing a security risk. Netease provides text-based and image-based CAPTCHAs, requiring users to click specific elements within an image. However, these CAPTCHAs can be challenging for users with physical impairments, affecting accessibility. Similarly, BeCAPTCHA-Mouse uses mouse dynamics and machine learning to distinguish between humans and bots, achieving 93% accuracy. However, it faces challenges such as variability in user behavior, high computational demands, accessibility issues for users with impairments, and the risk of advanced bots mimicking human movements [31]. These developments collectively represent a progressive response to the challenges posed by CAPTCHA farms.

**Fig 1:**
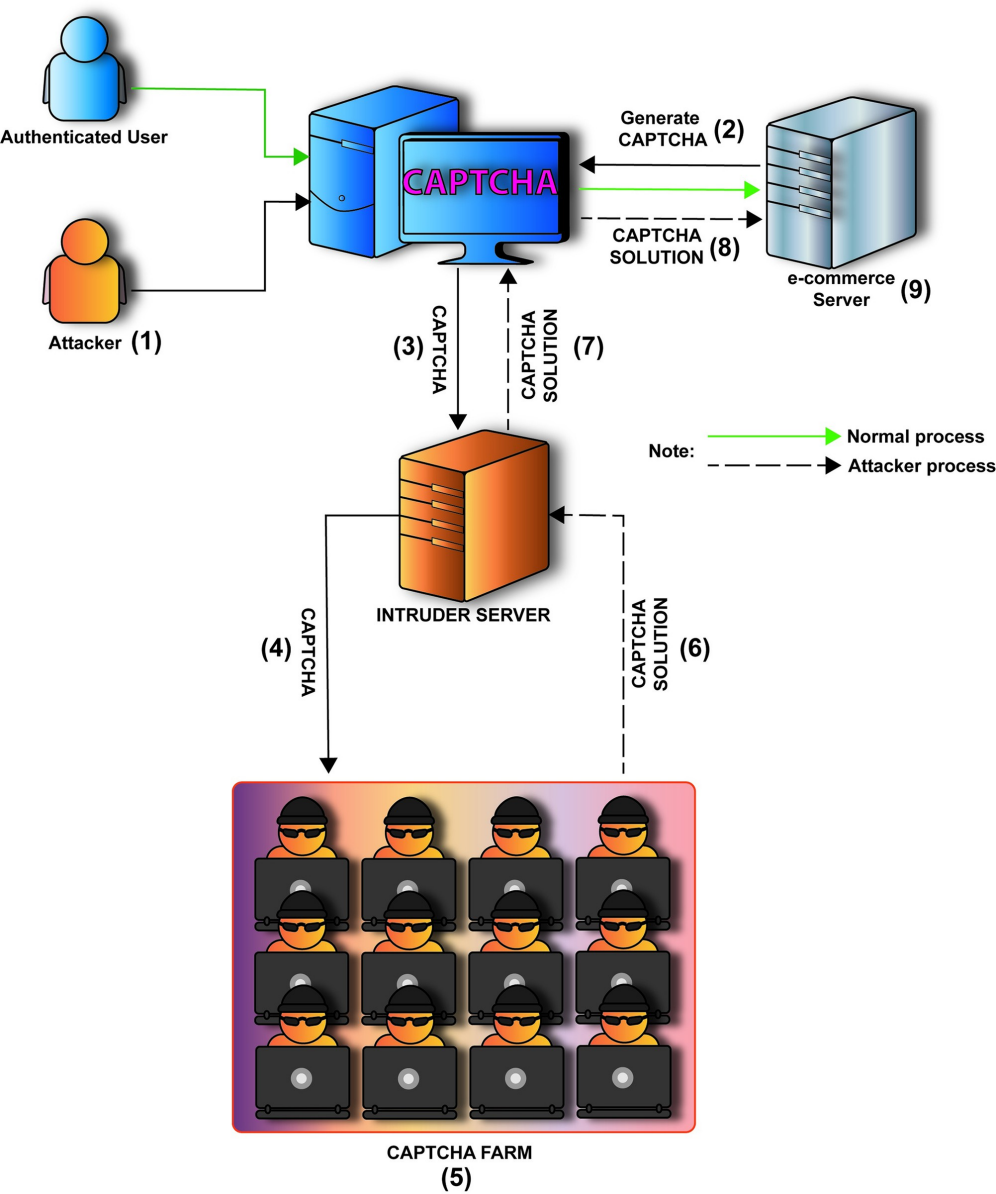
The model of a CAPTCHA farm attack.

Multi-factor authentication (MFA), which incorporates two additional security factors to identify users, is an alternative method for preventing CAPTCHA farm attacks. Users need a password and a random one-time password (OTP) to access the system, an example of MFA. In this case, the OTP will be sent to the user’s declared mobile device, and it must be entered on the login interface within a specified amount of time, such as 30 seconds [32, 33]. Therefore, it will be significantly more difficult for attackers to complete the login procedure without passing the OTP validation. The implementation of the iCAPTCHA system [10], which is an advanced CAPTCHA system designed to defend against third-party human CAPTCHA attacks, requires users to interact with a CAPTCHA test through a series of mouse clicks, which increases the statistical timing differences between legitimate users and human solvers. This method effectively detects human solver attacks with high accuracy and is user-friendly. However, it may pose challenges for users with physical impairments and require more computational resources traditional CAPTCHA systems. A notable characteristic of iCAPTCHA is the expiration mechanism, which ensures that the CAPTCHA is solved within the allotted time. Fig 1 illustrates the iCAPTCHA concepts.

There are currently a variety of factor combinations for MFA mechanisms, including some that use both biometric and physical factors and others that use physical locations and computing devices to acquire system access [34–36]. Consequently, the MFA can enhance the security layer on top of the CAPTCHAs because the MFA makes it much more difficult for bots and third-party attacks to bypass the security measures, even if they can solve the CAPTCHA.

Since the purpose of MFA is to prove the authenticity of the user, CAPTCHAs attempt to determine whether the user is a computer. In recent years, there have been efforts to integrate MFA and CAPTCHA so that human authentication can be confirmed simultaneously. For instance, by employing keystroke dynamics in text-based CAPTCHAs, the user can be identified while the CAPTCHA is typing [36–38]. In 2017, MFA technique, such as EYE-CAPTCHA [28] which employs three factors to identify a user and verify that they are not robots by combining gaze detection, eye movement, and the time taken to complete the CAPTCHA. This biometric-based CAPTCHA system achieves higher user satisfaction and security than traditional CAPTCHAs, effectively enhancing security and showing high reliability in preventing bot attacks. However, it requires specific hardware like webcams, poses accessibility challenges for some users, and involves a more complex verification process, which can lead to longer completion times. For a real-time system, in 2018, rtCAPTCHA, or real-time CAPTCHA [39] combines vocal and facial recognition with response time for solving puzzles. It integrates real-time user interactions to enhance security against automated attacks, achieving an overall accuracy of 89.2% in detecting human responses and ensuring high reliability by verifying facial and voice data. However, rtCAPTCHA may face challenges due to its complex verification process and the necessity for specific hardware, which can limit accessibility for some users. Additionally, sophisticated attackers could potentially exploit the system using advanced synthesis techniques. Similarly, BeCAPTCHA, a combination of MFA and CAPTCHA based on the mobile era, was proposed in 2020 [31]. This CAPTCHA method is implemented on mobile devices with an accelerometer installed. The user will be required to use a touch interface to solve a series of CAPTCHAs. The user’s behavior is analyzed and identified using accelerometer metrics during puzzle solving.

Some research has proposed using single-factor authentication with image-based CAPTCHAs, such as FATCHA [40], which requires users to make simple gestures like moving their head or blinking, which a webcam captures and the server recognizes. This system effectively distinguishes humans from bots and supports face recognition authentication. For gesture recognition, FATCHA achieves an accuracy rate of approximately 82%, and excluding more challenging gestures like smiling, the accuracy improves significantly, making it a robust and user-friendly security measure. However, FATCHA poses accessibility challenges for users without webcams or those with physical impairments, and it requires significant computational resources for effective gesture recognition.

In conclusion, the three CAPTCHA techniques, iCAPTCHA, rtCAPTCHA, and BeCAPTCHA, are trustworthy, quick, and secure and have been enhanced by adding MFA. With iCAPTCHA, it is quick and secure; FATCHA is quick and user-friendly, but security against sophisticated attacks may be compromised. In addition, EYE-CAPTCHA is a trustworthy, highly secure, and reliable mechanism that may be challenging to use. In addition, rtCAPTCHA is effective against sophisticated attacks, whereas BeCAPTCHA is also effective but requires additional sensors and technical knowledge.

The performance and limitations of each of each proposed solutions were evaluated using the standard performance metrics: precision, efficiency, security, usability, accessibility, physical protection, and protection against human attacks from other parties.

The CAPTCHAs mentioned above clearly have mechanisms that rely on biometrics and other behavioral characteristics, as well as additional security measures. Moreover, each has its own particular assets and weaknesses. The ratings range from moderate to high, indicating that all CAPTCHAs in Table 1 are secure against external attackers.

**Table 1:** Examples of the effectiveness of MFA-integrated CAPTCHAs.

CAPTCHA System	Year	Accuracy	Efficiency	Security	Usability	Accessibility	Protection Factors	Physical Protection	Strengths	Effectiveness Against 3rd Party Human Attack
iCAPTCHA [10, 40]	2011	High	High	High	Medium	Low	Image and text recognition	low	Efficient and secure	Moderate
FATCHA [40]	2015	High	High	Medium	High	High	Task-based	low	Accessible and efficient	Low
EYE-CAPTCHA [28]	2017	High	Medium	High	Low	Low	Gaze detection and eye movement	low	Secure and reliable	High
rtCAPTCHA [39]	2018	High	High	High	Low	Low	Mouse movement and behavioral analysis	Medium	Highly effective against sophisticated attacks	High
BeCAPTCHA [31]	2022	High	High	High	Medium	Medium	Mobile sensors and behavior analysis	low	Effective against sophisticated attacks	High

As shown in Table 1, although most CAPTCHAs use biometrics as a classification tool to differentiate between humans and bots, the interaction mechanism to acquire identification factors is quite complex and may not be user-friendly. In addition, the primary purpose of CAPTCHA remains to identify humans from machines. Therefore, biometrics should be more advantageous. Consequently, this study aims to develop a new type of CAPTCHA that can distinguish between humans and bots and perform authentication without requiring the user to recollect or perform additional steps during the log-in procedure.

As stated previously, the purpose of this paper is to propose a new CAPTCHA type called ProCAPTCHA. This ProCAPTCHA is a personalized CAPTCHA created based on biometric and profile-based information extracted from the individual’s personal profile. Thus, consumers will be protected from attacks by third parties. The anticipated results are that the proposed ProCAPTCHA will extend bot protection to the authentication system and resist to extant CAPTCHA-breaking techniques.

The following table compares various CAPTCHA methods developed over the years, highlighting their effectiveness in different aspects such as user-friendliness, accessibility, hardware requirements, biometric integration, and their capability to prevent various types of attacks. This comparison aims to provide an overview of CAPTCHA technologies evolution and their adaptability to emerging security challenges.

Table 2 presents a comprehensive comparison of various CAPTCHA methods developed from 2003 to 2020. Early methods by Von Ahn et al. (2003), Rusu and Govindaraju (2004), and Frank et al. (2018) focused on being user-friendly and effective against bots and brute force attacks. However, they lacked accessibility features and biometric integration.

**Table 2:** Comparison of various CAPTCHA methods and their effectiveness.

Year	2003 [4]	2004 [14]	2008 [15]	2014 [21]	2015 [40]	2016 [16]	2016 [24]	2017 [28]	2017 [25]	2018 [20]	2018 [23]	2019 [17]	2020 [31]
CAPTCHA method	Von Ahn et al.	Rusu and Govindaraju	Chow et al.	Google’s "No CAPTCHA reCAPTCHA"	De Marsico et al. (FATCHA)	NuCAPTCHA	Hupperich et al. (SensorCAPTCHAs)	Siripitakchai et al. (EYE-CAPTCHA)	Pedometric	Frank et al.	Guerar et al. (CAPPCHA)	Kim S, Choi S (DotCHA)	Acien et al. (BeCAPTCHA-Mouse)
Attributes													
User-friendly	**✓**		**✓**	**✓**	**✓**	**✓**	**✓**	**✓**	**✓**		**✓**	**✓**	**✓**
Accessibility				**✓**	**✓**								
Hardware requirement		**✓**				**✓**	**✓**	**✓**	**✓**	**✓**	**✓**	**✓**	**✓**
Biometric integration								**✓**					**✓**
Effective against bots	**✓**	**✓**	**✓**	**✓**	**✓**	**✓**	**✓**	**✓**	**✓**	**✓**	**✓**	**✓**	**✓**
Effective against CAPTCHA farm				**✓**		**✓**	**✓**	**✓**	**✓**		**✓**	**✓**	**✓**
Ease of implementation	**✓**			**✓**		**✓**	**✓**	**✓**	**✓**		**✓**	**✓**	**✓**
Cost of implementation	**✓**			**✓**		**✓**	**✓**	**✓**	**✓**		**✓**	**✓**	**✓**
User experience			**✓**	**✓**	**✓**	**✓**	**✓**	**✓**	**✓**		**✓**	**✓**	**✓**
Effectiveness in Preventing Automated Script Attacks	**✓**	**✓**	**✓**	**✓**	**✓**	**✓**	**✓**	**✓**	**✓**	**✓**	**✓**	**✓**	**✓**
Effectiveness in Preventing Phishing Attacks						**✓**							
Effectiveness in Preventing Brute Force Attacks						**✓**	**✓**	**✓**	**✓**		**✓**	**✓**	**✓**

Chow et al. (2008) introduced a user-friendly method that was effective against bots but did not address accessibility or advanced threats. Google’s "No CAPTCHA reCAPTCHA" (2014) marked a significant improvement by being user-friendly, accessible, and highly effective against various attacks, including bots, CAPTCHA farms, automated scripts, phishing, and brute force attacks.

In 2015, De Marsico et al. (FATCHA) combined user-friendliness and accessibility with effectiveness against bots. NuCAPTCHA (2016) and Sensor CAPTCHAs by Hupperich et al. (2016) continued this trend, requiring minimal hardware and highly effective against various attack methods.

EYE-CAPTCHA by Siripitakchai et al. (2017) integrated biometric data, enhancing its effectiveness against bots and various attacks while requiring specific hardware. Pedometric CAPTCHA (2017) and Guerar et al.’s CAPPCHA (2018) focused on user-friendliness and effectiveness against bots and brute force attacks, utilizing mobile sensors and cognitive puzzles, respectively.

DotCHA (2019) by Kim S and Choi S and BeCAPTCHA-Mouse (2020) by Acien et al. introduced advanced methods that were user-friendly, hardware-requirement-specific, and highly effective against a broad spectrum of attacks, with BeCAPTCHA-Mouse also integrating biometric data. These recent methods represent the ongoing evolution of CAPTCHA technologies towards greater security and user adaptability.

#### Problem statement

With an increasing reliance on online platforms for various daily activities, the significance of security measures cannot be overemphasized in the modern digital age. CAPTCHAs have emerged as one of the most essential protections against malicious automated activities, serving to distinguish between humans and bots. Recent developments and sophisticated assault methods, such as the use of CAPTCHA farms, pose significant challenges to the status quo of online security despite their historical efficacy. In addition, as these systems endeavor to become more complex to counteract emergent threats, they inadvertently introduce an abundance of usability issues. Consequently, there is an immediate need to examine and resolve the current CAPTCHA design and implementation flaws. In light of this, the following challenges are identified:

- **CAPTCHA Farm attacks:** Even though CAPTCHA systems are intended to ward off bots, the emergence of CAPTCHA farms has reversed the equation. These farms, while potentially beneficial for legitimate purposes, have become a tool for malicious actors. Exploiting human solvers, they bypass CAPTCHA systems to execute nefarious activities such as creating fake accounts, dispatching spam, or instigating automated attacks.
- **User-Friendly Problems:** The modern CAPTCHA designs often end up being a double-edged sword. While they might be complex enough to deter automated bots, this complexity usually translates to a cumbersome experience for genuine users. Problems arise when CAPTCHAs incorporate unfamiliar equipment, erroneous timestamps, or rely on user actions that might be discomforting. Additionally, implementing certain CAPTCHAs requires tools that are expensive and might lack long-term sustainability.
- **Unauthorized access:** A critical flaw in the current CAPTCHA system is its focus on differentiating humans from bots, neglecting the essential aspect of identity verification. Even if a CAPTCHA is solved by a human, it doesn’t guarantee that the solver is the legitimate user or that it’s a third-party attacker, leaving a considerable gap in user authentication.
- **Economic and Longevity Concerns**: CAPTCHA depend on costly tools or devices, making them less accessible to a broader audience. Moreover, these tools and devices are generally updated from time to time, which can affect the relied CAPTCHAs. Thus, the sustainability of such CAPTCHAs cannot be maintained.
- **Latency and Performance Issues**: Since there are some image-based CAPTCHAs that use various images and graphics to interact with a user, these types of CAPTCHAs can cause a high latency while loading for the bot-verification process. So, the performance of the bot verification is slow and may easily be broken by a third party. Another unexpected issue is that these CAPTCHAs might not be able to be displayed on an old user’s device. Thus, the authorized user cannot access the required system due to a failure in the bot verification process.

To overcome the challenges above, various research areas are required, including cybersecurity, user experience design, cognitive science, and accessibility studies. So, this paper aims to combine all compulsory fields to create a new type of CAPTCHA, namely, ProCAPTCHA.

#### Aims and objectives

*Aims*. The research aims to create a new type of CAPTCHA, which is a bot verification against the CAPTCHA farm attack combined with an authentication technique so a human can be identified as a real and authenticated user at once. The proposed CAPTCHA, called ProCAPTCHA, is a text-based CAPTCHA that will be generated based on all features mentioned previously: cybersecurity, user experience design, cognitive science, and accessibility studies. The primary technique of ProCAPTCHA relies on behavioral biometrics, which will assess its effectiveness in preventing unauthorized access by CAPTCHA farms.

The significant characteristic of ProCAPTCHA is that it is generated using an individual’s profile information. The performance metrics are accuracy, efficiency, security, usability, accessibility, physical protection, and defense against human attacks from third parties.

*Objectives*.

1. Identify characteristics, techniques, and impacts of CAPTCHA Farm. This objective seeks to explore the intricacies of CAPTCHA Farms. By understanding their characteristics and techniques, the study intends to unravel how these farms operate, their methodologies, and the technologies they deploy. Furthermore, by identifying the direct and indirect impacts of these farms on online platforms, the objective provides a holistic view of the challenges they present.
2. Select a suitable biometric to be applied within the ProCAPTCHA. The objective is to identify a biometric that precisely corresponds with the ProCAPTCHA system’s security demands and user experience requirements. This phase involves evaluating the feasibility, accuracy, and dependability of the available biometrics, from single biometrics to cross-sectional associations between physical behavior patterns, in relation to CAPTCHA authentication.
3. Identify common but significant characteristics of any authenticated users. To make ProCAPTCHA efficient, it’s essential to understand the consistent features or patterns that real authenticated users exhibit. These patterns could be in their behavior, interactions, or other discernible traits during CAPTCHA verification. By understanding these, a CAPTCHA system can be more intuitive and user-centric while maintaining robust security.
4. Determine the classification model for classifying an authenticated non-bot user. The essence of this objective is to develop a predictive model that can distinguish between legitimate human users and bots or unauthorized users. Using collected data, the research will employ machine learning or classification techniques to train a model that classifies user interactions. The accuracy and reliability of this model are crucial, as it determines the effectiveness of the ProCAPTCHA system.
5. Determine the performance of the obtained classification model based on the performance metrics mentioned previously. After developing the classification model, it’s imperative to evaluate its performance. This objective emphasizes evaluating the model’s performance against predefined metrics like accuracy, efficiency, security, and more. Through rigorous testing and validation, the study seeks to ensure that the classification model meets or surpasses the expected standards, thereby solidifying its place in the ProCAPTCHA system.

Based on the aims and objectives above, the details of the contributions are as follows.

#### Contribution

As mentioned previously, the new ProCAPTCHA is generated by combining various fields to protect the CAPTCHA farm from attacks and unauthorized access. Therefore, the contributions of this work are as follows:

1. A biometric called keystroke dynamics has been applied for cybersecurity protection.
2. The text-based CAPTCHAs that most cyber-users are familiar with are selected. This satisfies the user experience property.
3. The common but significant characters of each individual authenticated user are applied so users can be recognized easily. This satisfies the cognitive science property.
4. The ProCAPTCHA contains all black characters, so every typeable person, including colorblind people, can be involved. This satisfies the accessibility study property.
5. ProCAPTCHA can detect and identify a real and authenticated human user correctly.

### Expected outcomes

As a consequence of the above contributions, the expected outcomes of this research can be listed below.

1. **Introduction of ProCAPTCHA System**: A significant contribution is the development and proposal of the ProCAPTCHA system. This innovative system employs biometric and profile-based data to craft individualized CAPTCHAs, potentially revolutionizing the CAPTCHA paradigm by ensuring enhanced security while catering to the individual user’s experience.
2. **Enhancement of Identity Verification:** The research contributes novel methodologies within the ProCAPTCHA systems that can strengthen identity verification. These advanced techniques ensure a dual focus: distinguishing humans from bots and authenticating the genuine identity of users.
3. **Comprehensive Usability Evaluation:** The study conducts an extensive usability assessment of existing ProCAPTCHA designs, shedding light on areas of disability for genuine users. This comprehensive evaluation of ProCAPTCHA serves as a blueprint for maintaining a balance between security and user-friendliness.
4. **Perspectives on Economy and Longevity:** This implementation of ProCAPTCHA concerned a critical assessment of the economic feasibility and long-term viability of existing CAPTCHA tools. Based on this analysis, the researchers can claim that ProCAPTCHA is both cost-effective and sustainable, providing valuable insights for businesses.

## 3. System model

### 3.1 CAPTCHA farms model

CAPTCHA farms and the services they provide have drastically altered the dynamics of online security and bot mitigation. By harnessing the power of human solvers, these farms enable malicious actors to bypass what was once a formidable obstacle in the form of CAPTCHAs. A deeper dive into the workings and implications of CAPTCHA farms based on the provided details is elaborated below:

- Cost-Effective Bypass Mechanisms of CAPTCHA Farms:

CAPTCHA farms, predominantly based in regions with low labor costs, have made it commercially viable for attackers to bypass security challenges. The inexpensive nature of these services, which can be as low as $1–3 for every 1,000 CAPTCHAs, depending on the CAPTCHA type, poses a significant challenge to online platforms that depend on CAPTCHAs for security [9, 22].

- The Lifecycle of a CAPTCHA Farm-Assisted Bot Interaction:

This hypothetical attacker model describes a scenario (1), (2), (3), and (4), as depicted in the Fig 1, in which a person or group attempts to circumvent CAPTCHA security measures by combining human intervention, machine learning, and economic exploitation. In this model, an attacker with intermediate to advanced skills seeks unauthorized access to websites or services by delegating CAPTCHA-solving tasks to a network of human employees.

Details of CAPTCHA farm processing are displayed in Fig 1 and described as follows.

1. Reconnaissance: Before the actual attack, an attacker conducts a preliminary reconnaissance on the target website. This initial survey helps identify CAPTCHA as one of the site’s defense mechanisms.
2. Initial Connection: When the attack commences, the bot attempts to access the target website. The site, in response, presents a CAPTCHA challenge, aiming to verify the authenticity of the requester.
3. CAPTCHA Capture: On encountering the CAPTCHA, the bot captures a clear image of the challenge. This image is crucial as it is what the human solvers will interpret and solve.
4. Request to CAPTCHA Farm: Using CAPTCHA farm business dedicated API, the bot sends the captured CAPTCHA image for resolution. The service takes over from this point, distributing the challenge to one or more of its human solvers, such as the CAPTCHA’s public key and the domain name, to the farm.
5. Human Solution: These human solvers from the CAPTCHA farm are assigned the task, and within a matter of seconds (typically 30–45), the CAPTCHA is solved.
6. CAPTCHA Resolution: The bot receives the solution and sends back to the bot using the same API.
7. Bot’s Submission: Armed with the correct solution, the bot submits the resolved CAPTCHA back to the website.
8. CAPTCHA Clearance: The bot then uses this token to clear the CAPTCHA challenge, allowing it to proceed with its intended task.
9. Website’s Misjudgment: The website, receiving a correct CAPTCHA solution, wrongly assumes the requester is human. Consequently, it grants access, allowing the bot to proceed with its intended task.
  - Implications for Online Security

The existence and efficiency of CAPTCHA farms underscore the need for evolving online security measures. While CAPTCHAs were initially designed as a deterrent against automated attacks, the human-powered solutions offered by CAPTCHA farms have made them less effective. As a result, online platforms must reconsider their security strategies, possibly integrating multi-factor authentication or other advanced measures, to ensure they remain protected against increasingly sophisticated threats.

### 3.2 System model

In the realm of cybersecurity and user authentication, traditional CAPTCHA systems have been long-standing guardians that have distinguished humans from automated bots. The biometric authentication has expanded beyond traditional physiological measures to incorporate behavioral traits. A notable study proposed an innovative approach to this, suggesting that personalized, static information, such as one’s name, surname, email, and phone number, could serve as consistent biometric authentication factors [41]. The premise rested on the unique patterns and rhythms individuals exhibit when inputting this static data—patterns that remain relatively consistent over time. These unique typing dynamics, from the speed and rhythm to the pressure exerted on touch screens, formed a distinct biometric signature for each individual. Leveraging the insights from this foundational research, this research aims to integrate these consistent biometric factors into the design of a new CAPTCHA system. Instead of relying solely on the traditional challenges that CAPTCHAs present, the proposed system seeks to intertwine user authentication with bot-human differentiation, drawing from the biometric groundwork laid by the aforementioned study.

**Fig 2:**
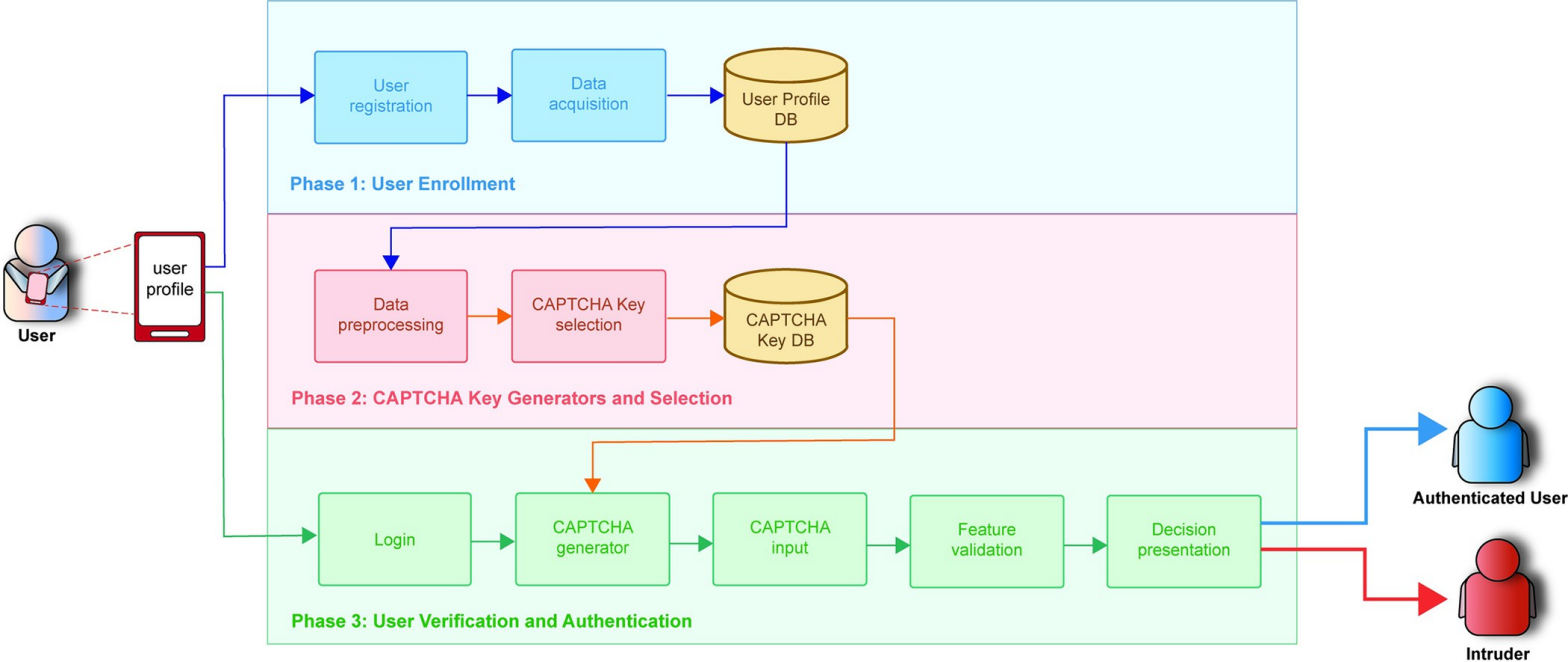
System architecture for authentication classification.

The proposed methodology can identify a user who uses a mobile device. As mentioned previously, this proposed system is its infusion traditional CAPTCHA challenge, as Text-based CAPTCHA that includes the user’s keystroke dynamics based on the user’s profile data. The new system of CAPTCHA offers a dual-fold advantage: bot deterrence and user authentication. This dynamic system is strategically bifurcated into three primary phases: User Enrollment, CAPTCHA Key Generator and Selection, and User Verification and Authentication phases. Fig 2 explains the architecture of the proposed system.

The architecture depicted in Fig 2 details a three-phase process for user authentication using CAPTCHA as a verification tool. The process commences with the User Enrollment phase, where individuals provide foundational information to form or update their user profile via the user registration process. This profile is further refined with additional behavioral information gathered during the data acquisition process and is initially processed and stored in the User Profile database. Proceeding to the CAPTCHA Key Generators and Selection phase, this information is refined through Data Preprocessing to extract unique behavioral patterns, which then inform the selection of a distinct CAPTCHA Key. This key facilitates the creation of a bespoke CAPTCHA challenge, which is specifically designed for each user and stored in the CAPTCHA Key database. The concluding phase, User Verification, and Authentication, involves a series of steps where users must log in, and then the module CAPTCHA generator randomly retrieves keys from the CAPTCHA Key database to generate a CAPTCHA challenge. Afterwards, the user must enter this CAPTCHA via the CAPTCHA input module within the exact time limit, where all keystroke dynamics are compared with the values in the CAPTCHA Key database based on the selected keys performed in the Feature verification module. The outcome from the Feature validation is shown in the Decision presentation module, which is either an intruder or an authenticated user.

The system is strategically divided into three primary phases: **User Enrollment**, **CAPTCHA Key Generator and Selection**, and **User Verification and Authentication**.

The architecture depicted in Fig 2 outlines a comprehensive three-phase process for user authentication using CAPTCHA as a verification tool:

1. **User Enrollment Phase**:
  ○ The process begins with the User Enrollment phase, where individuals provide foundational information to create or update their user profiles through the user registration process.
  ○ This profile is further enhanced with additional behavioral data gathered during the data acquisition process.
  ○ The collected information is then processed and stored in the **User Profile database**.
2. **CAPTCHA Key Generator and Selection Phase**:
  ○ In this phase, the stored profile data undergoes further refinement through a **Data Preprocessing** process, which extracts unique behavioral patterns.
  ○ Based on these patterns, the system selects a distinct CAPTCHA Key tailored to the user, facilitating the creation of a personalized CAPTCHA challenge.
  ○ This CAPTCHA Key is then stored in the **CAPTCHA Key database**.
3. **User Verification and Authentication Phase**:
  ○ During this final phase, users must log in to initiate the verification process.
  ○ The **CAPTCHA Generator module** randomly retrieves keys from the CAPTCHA Key database to generate a CAPTCHA challenge.
  ○ Users are required to enter this CAPTCHA through the **CAPTCHA Input module** within a specified time limit.
  ○ The system compares the user’s keystroke dynamics with the values stored in the CAPTCHA Key database using the **Feature Verification module**.
  ○ Based on the outcome of this verification, the **Decision Presentation module** will either authenticate the user or flag them as an intruder.

## 4. Propose system and methodology used

ProCAPTCHA stands as a novel authentication system that merges traditional CAPTCHA mechanisms with the unique typing dynamics of individuals. This system is designed to verify authenticated users not just by what they input (i.e., the correct CAPTCHA response) but also by how they input it (i.e., their keystroke dynamics). It operates on two distinct phases–the User Enrollment Phase (UEP) and the User Verification Phase (UVP)–each composed of several critical modules.

**Fig 3:**
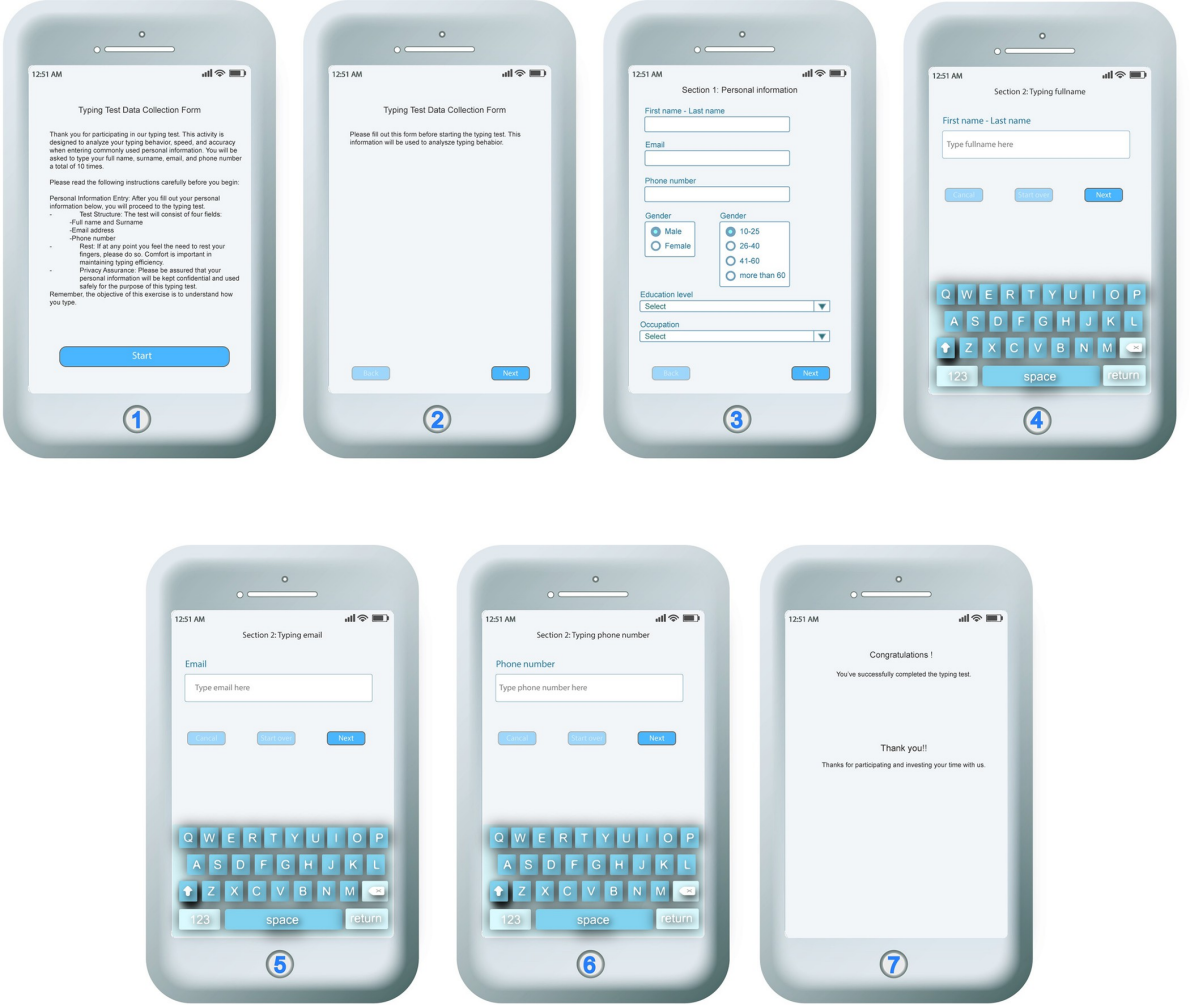
Different stages of data collection forms.

In the Fig 3 illustrates a sequence of screens from a mobile web application designed to collect typing data for a system called ProCAPTCHA. The process begins with an introductory screen that informs participants about the purpose of the test and guides them on how to proceed. Following the introduction, there’s a screen with instructions emphasizing the need for careful data input. Next, participants fill in their personal information, including their name, email, phone number, and demographic details. Subsequent screens are dedicated to the actual typing test, where users input their name, email, and phone number, with the system recording their keystroke dynamics to form a unique typing pattern profile. This process is repeated multiple times for personal unique typing. The final screen in the figure serves as a confirmation to participants that they have finished the data collection process. This multi-step procedure is essential for building a behavioral biometric profile for each user, which ProCAPTCHA can use to verify the identity based on typing patterns, enhancing security measures.

The following sections describe all phases related to the proposed architecture, as shown in Fig 2.

### 4.1 User Enrollment Phase (UEP)

Every user starts at this step, which is made up of two processes: the data collecting process and the user registration procedure. Before going on to the next step of creating a suitable CAPTCHA character for each individual, the goal of this phase is to gather the personal traits of each user. The User Profile database houses the information gathered during this stage.

This phase’s two procedures are accomplished via a web application designed to be used with iPhone 7 or later models. The experiment’s target population consists of volunteers who are between the ages of 18 and 21 and have at least three years of experience using a touchscreen on a mobile device. Only forty-five participants could participate due to COVID-19 restrictions.

HTML and PHP were used in the development of the web application, which is essential for data entry. JavaScript was used to handle data collecting. It uses the iPhone’s Chrome browser to function. The entire name, last name, email address, and phone number are the only required user data that must be provided using the iPhone’s touchscreen. Keystroke feature analysis requires the capturing of keyboard events, such as key press and pressure, which is what this application does.

#### 4.1.1 User registration process

Upon accessing the website, the data collection procedure unfolds as depicted in Fig 3, where participants are prompted to furnish demographic information, including age, gender, educational level, and occupation, as shown in screen 3 of Fig 3. After finishing this process, the personal characteristics will be collected via the data acquisition process as described below.

#### 4.1.2 Data acquisition process

In this process, each new participant must enter static details via a virtual keyboard, as shown in Fig 3; these are their name, surname, email, and phone number. Throughout this procedure, the platform logs all keystroke dynamics, which encompass dwell time, flight time, and typing speed.

The static details that each user must type through screens 4, 5, and 6 of Fig 3 are their full name, surname, email, and phone number. This data must be entered repeatedly, amounting to a total of ten times. Then, the iOS system proceeds to log the timing of each keystroke. This collected keystroke data is compiled and transmitted to a user profile database afterward. This database, the user profile database, can utilize the uniqueness of individual users. The typing patterns, such as speed and rhythm, can be scrutinized and used to assess factors to identify the user. Therefore, the behavioral biometric profile for user authentication can be created.

#### 4.1.3 User profile database

To keep usable data for further usage, the data collected from the data acquisition process is stored in the User Profile database. Those data include the character value, keystroke timings, fingertip location and radius, timestamp, and demographic details such as gender, age, occupation, and education level.

### 4.2 CAPTCHA key generator and selection

After passing the User Enrollment phase, the next step is to generate a suitable CAPTCHA and test the efficiency of user identification. This phase is named the CAPTCHA Key Generator and Selection phase. Under this phase, there are three processes to be performed: the Data Preprocessing process and the CAPTCHA Key Selection process. In addition, there is one database involved, the CAPTCHA Key Database. Details of every element are described below.

#### 4.2.1 Data preprocessing process

For data preprocessing process, or so called as data cleansing, is the collected keystroke data undergoes preprocessing where irrelevant features such as special characters are removed, and outliers are filtered out. This ensures the data’s integrity for further analysis.

This process receives data from the User Profile database, where individual characters and their corresponding key events are stored. The characters are stored as key codes, while key events like key up, key down, and key press are logged whenever a key is interacted with on the virtual keyboard. These events are used to calculate keystroke features and are stored as parameters in the CAPTCHA Key database (see Table 3 for calculations).

**Table 3:** The formula for calculating keystroke values.

Experiment Feature	Abbreviations	Formula
Dwell time	Dw	*Dw*_*i*_ = *R*_*i*_−*P*_*i*_
Interval time	In	*In*_(*i*,*i*+1)_ = *P*_*i*+1_−*R*_*i*_
Latency time	La	*La*_(*i*,*i*+1)_ = *R*_*i*+1_−*P*_*i*_
Flight time	Fl	*Fl*_(*i*,*i*+1)_ = *P*_*i*+1_−*P*_*i*_
Up to up time	Up	*Fl*_(*i*,*i*+1)_ = *R*_*i*+1_−*R*_*i*_

*note: *i* = character# *i*, *R*_*i*_ = time to release *i*, *P*_*i*_ = time of pressing *i*

**Fig 4:**
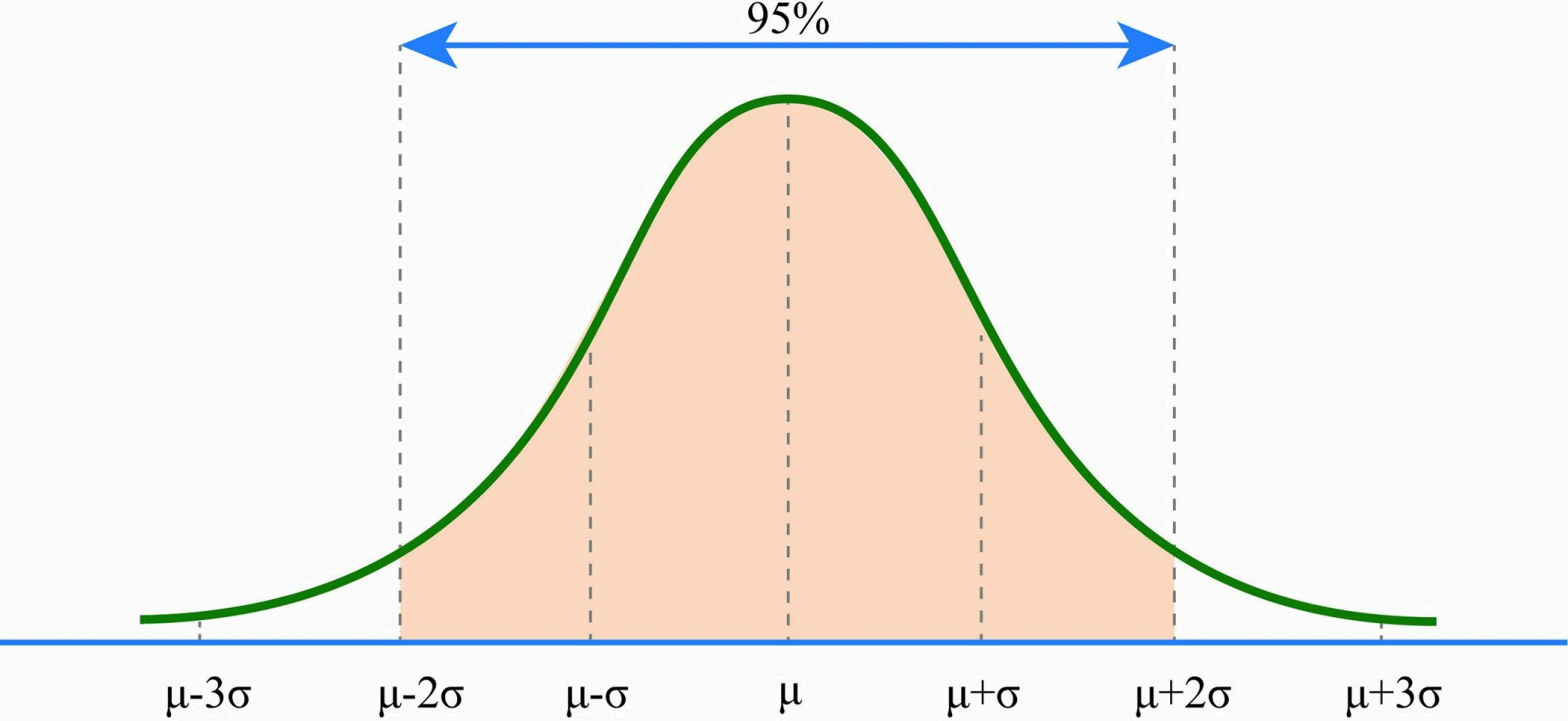
The area under 2 standard deviations using the Empirical Rule [42].

**Outlier Detection:** Before data are transferred to the CAPTCHA key selection process, it must pass the validation process to ensure consistency and mitigate external typing influences. Outliers are removed using the Empirical Rule, with 95% of data expected to fall within the range of (μ ± 2σ) (see Fig 4). The values for μ and σ are derived using Formulas (1) and (2).

Where:

$$\mu =\frac{\sum_{i=1}^{n}x_{i}}{N}$$ (1)

$$\sigma =\sqrt{\frac{\sum_{i=1}^{n}\left(x_{i}-\bar{x}\right)}{n-1}}$$ (2)

#### 4.2.2 CAPTCHA key selection process

The CAPTCHA key selection process is a critical step in establishing a robust authentication system. This process commences by analyzing the frequency of character use within a given dataset, typically derived from the user’s typing behavior logged in the User Profile database. The primary objective is to identify and select characters and character pairs (digraphs) that the user inputs most frequently when interacting with the system.

The concept of using frequency analysis is based on the belief that people are always familiar with their name, surname, email, and personal phone number rather than the setup passwords. Thus, the most used characters and digraphs from their personal information can become the prime candidates for indicating a user. Thus, these selected characters and digraphs can be used to generate a unique CAPTCHA key, and this unique CAPTCHA key can authenticate a user from an intruder or CAPTCHA farm.

In conclusion, the rationale behind this selection is twofold:

- Personalization: By choosing characters and digraphs that the user types frequently, the system ensures that the CAPTCHA challenge is personalized and, in theory, easier for the user to solve due to familiarity with the characters; the letter is the user’s name, email address, and phone number, which they type frequently, which increases their familiarity with typing them.
- Security: Personalization adds a layer of security because the CAPTCHA challenges become user specific. An impersonator would not only need to solve the CAPTCHA but would also need to know the specific characters and digraphs that the legitimate user types frequently, which is a much harder task without access to their typing data. Once the frequency analysis is complete, the system ranks the single characters and digraphs by their frequency scores. The top ten single characters and the top five digraphs are then earmarked as the user’s CAPTCHA keys. These selected keys, along with the associated keystroke dynamics, such as the speed of typing, dwell time, and flight time between keys, are meticulously recorded in the CAPTCHA Key database.

To elucidate with a practical example, consider the sample profile information as:

***Name*:** John Doe

***Email*:** johndoe@example.com

***Phone number*:** 123-456-7890

The system will break down these input strings into the constituent single characters and digraphs as follows:

***Constituent single characters*:** j, o, h, n, d, e, x, a, m, p, l, c, 1, 2, 3, 4, 5, 6, 7, 8, 9, 0

***Digraphs*:** jo, oh, hn, do, oe, jo, oh, hn, nd, do, oe, ex, xa, am, mp, pl, le, co, om, 12, 23, 34, 45, 56, 67, 78, 89, 90

The system then performs a frequency analysis to ascertain how often each character and digraph appears within the user’s input across all collected data. After tabulating the frequencies, the top ten single characters and the top five digraphs are selected.

Thus, from the example above, the selected characters are "o", "n", "e", "1", "2", "3", "4", "5", "6", and "j" because their occurrences are the highest amongst other characters. Similar to the single characters, under the considered condition, the list of top-five digraphs is "jo", "oh", "hn", "12" and "23".

After obtaining the selected characters and digraphs, these outcomes will be stored in the CAPTCHA Key database, including their associated typing dynamics.

#### 4.2.3 CAPTCHA key database

**Fig 5:**
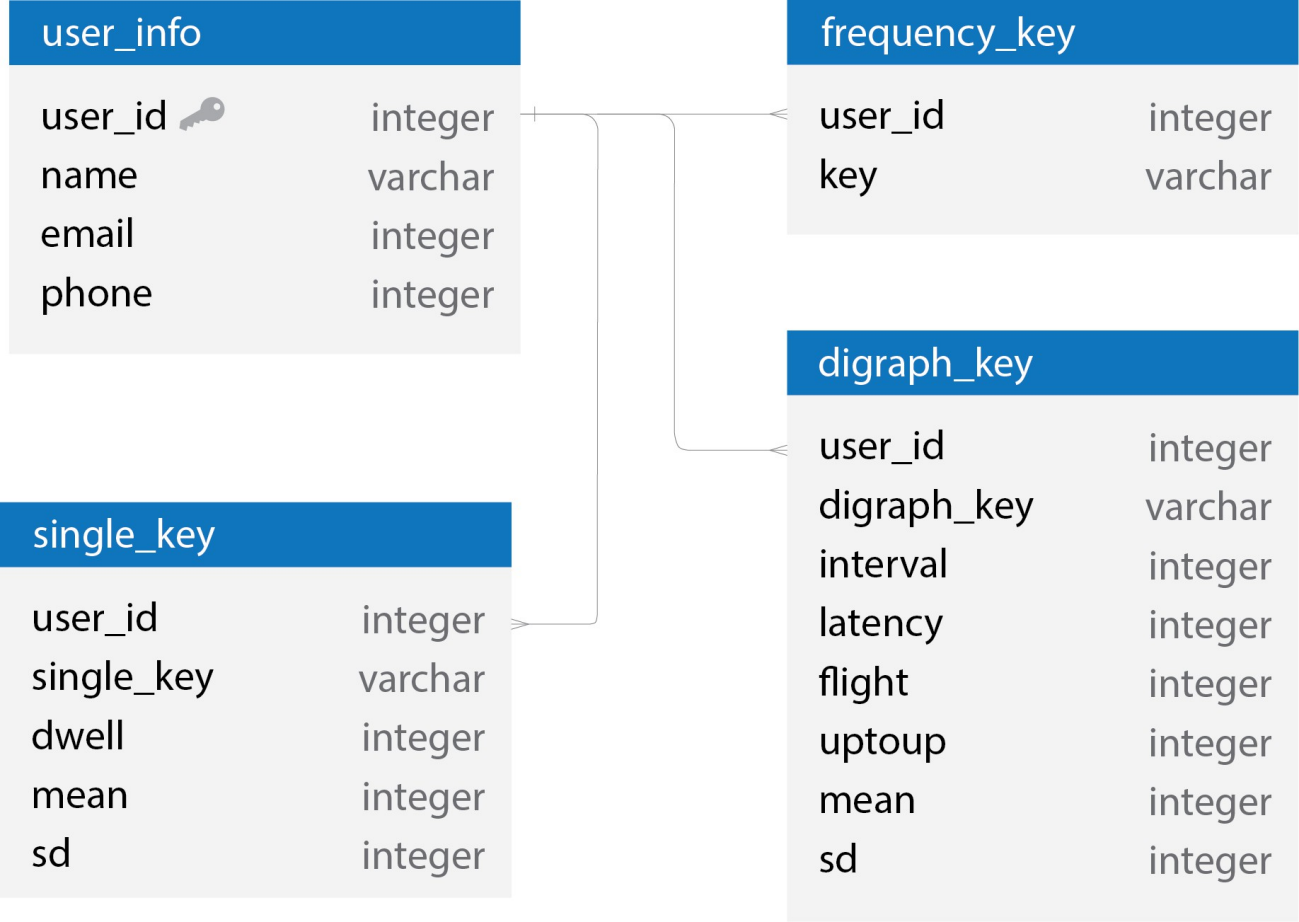
A database schema for a CAPTCHA key database.

The database contains sophisticated and significant information that is utilized to improve user authentication and verification. According to the outcomes of the CAPTCHA Key Selection process, 15 characters for each user, consisting of the top-ten non-redundant single characters and the top-five digraphs, are collected and recorded in this database. Moreover, other outcomes from the enrollment phase, which are keystroke dynamics between enrolling the user profile, are real-time captured and stored as significant personal values related to all 15 selected characters, as mentioned previously. These time values are dwell time (Dw), interval time (Iv), latency time (La), flight time (Fl), and up-to-up time (Up). Moreover, the mean time ($\bar{x}$) and standard deviation (*σ*) for each character’s time are calculated. For example, every single character in the CAPTCHA Key database contains the mean time of its dwell time, not interval time (Iv), latency time (La), flight time (Fl), and up to up time (Up), while every digraph in the CAPTCHA Key database contains the mean time of its interval time (Iv), latency time (La), flight time (Fl), and up to up time (Up), and not dwell time. Fig 5 shows the designed schema of the CAPTCHA Key database that can enhance user authentication for each user.

As in Fig 5, the schema presents several tables, such as **user_info**, **single_key**, **frequency_key**, and **digraph_key**, each containing different fields. The **user_info** table seems to be storing basic user information, such as **user_id**, **name**, **email**, and **phone**. The **single_key** table records details about individual keystrokes, with fields for the **user_id**, the key itself (**single_key**), **dwell** time (how long the key is pressed), **mean** (average) times, and **sd** (standard deviation), which suggests measurement of typing consistency. The **frequency_key** table, although not entirely visible, seems to be related to the frequency of key usage. The **digraph_key** table contains fields that are specifically about digraphs (two-character combinations), like **digraph_key**, **interval**, **latency**, **flight**, **uptoup**, **mean**, and **sd**. This table does not include dwell time, unlike the **single_key** table, but it does include other time measurements between key presses. The relationships between tables indicate that they are linked via the **user_id** field, ensuring that keystroke dynamics can be associated with the correct user profile.

By conclusion, the data stored in the CAPTCHA Key database are, for each user profile, 15 characters, 10 non-redundant single characters, and 5 digraphs and their related time values with their means and standard deviations. Consequently, these data can support the creation of ProCAPTCHA, which can maintain personal security and authenticity as needed.

This CAPTCHA Key information is then utilized in the next step to generate a customized CAPTCHA for each user, named ProCAPTCHA. The ProCAPTCHA is intricately designed to align with the user’s unique typing patterns and characteristics derived from the data stored in the database. It reflects the user’s individual typing behavior, ensuring a personalized and secure interaction in the Feature verification module during the User Verification and Authentication phase.

### 4.3 User verification and authentication phase

During the User Verification and Authentication phase, the primary goal is to authenticate individuals before they are granted access to the system. This phase encompasses five key modules, depicted in Fig 2, with each module’s function detailed below.

**Fig 6:**
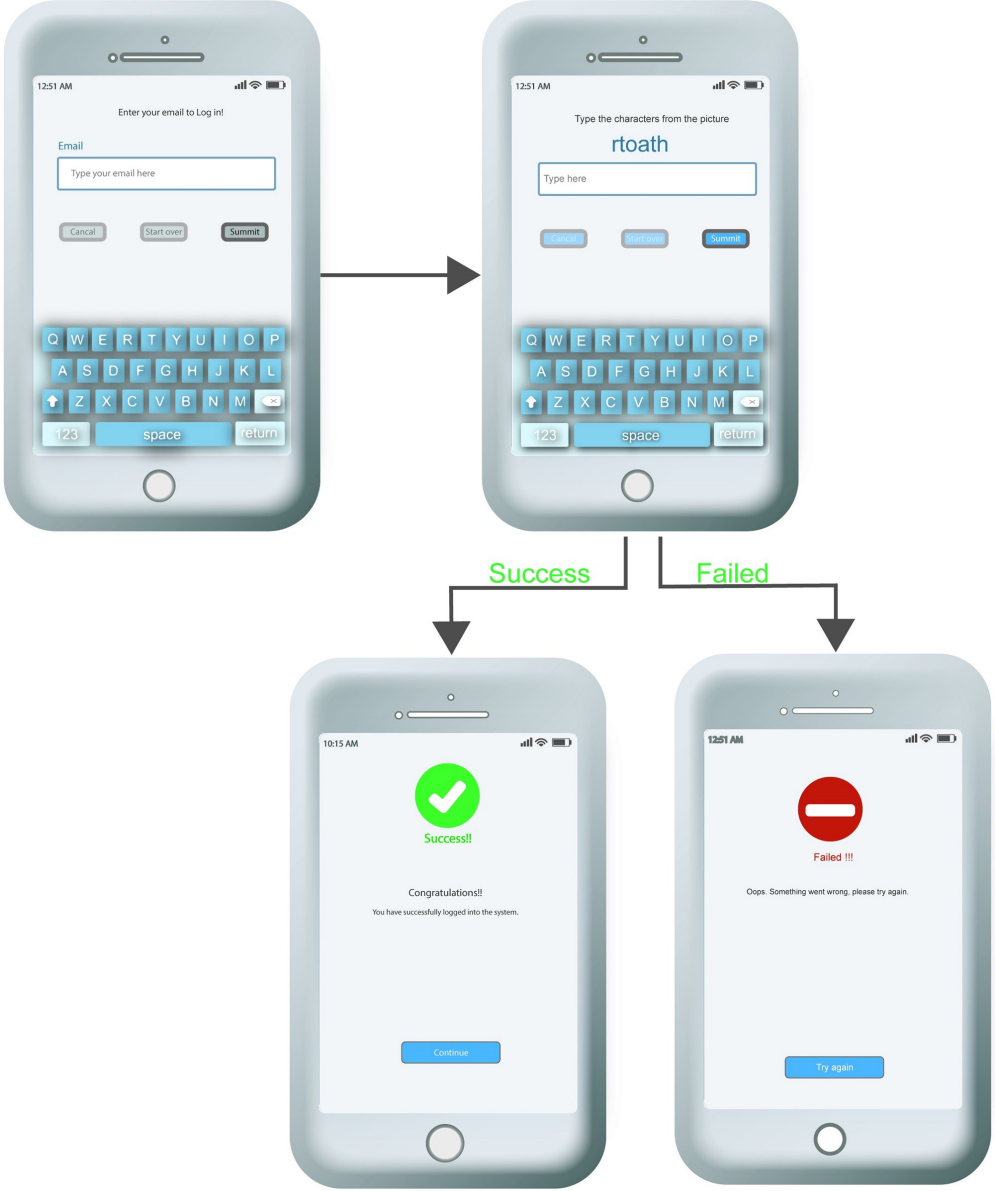
User verification and confirmation screens.

In Fig 6, a depiction of a user verification and authentication procedure has been unfolded in two primary stages. Initially, the user is required to enter their email address, which serves as a form of identification for their account. Following this, the user encounters a CAPTCHA test. This test requires the user to type in a set of characters that are displayed on the screen, a step designed to ascertain that the user is a legitimate person rather than an unauthenticated person. Successful navigation through these stages leads to either one of the last two screens. If the user is notified that the verification has been successfully passed, it will grant them access to their account. Otherwise, the user is given the option to ’try again’.

All processes mentioned above are performed by the following modules as described below.

#### 4.3.1 User Login module

Users are prompted to enter their email address, initiating the verification phase. The initial step in the User Verification and Authentication phase is the Login module, where users are required to utilize their email addresses to log into the system. This email is cross-referenced with the CAPTCHA Key database that is used to generate a Text-based CAPTCHA tailored to the user. The CAPTCHA Generator module is responsible for this task and operates as follows.

#### 4.3.2 CAPTCHA Generator process

The CAPTCHA Generator process is a sophisticated mechanism for user authentication that creates personalized CAPTCHA values. It commences by extracting specific characters and digraphs from the CAPTCHA Key database, which contains the most frequently used keystrokes by the user. These elements are then assembled into a distinctive CAPTCHA sequence, intended to verify the user’s identity.

In the CAPTCHA system, a CAPTCHA challenge is presented to the user as a sequence of six slots. These slots are the placeholders where characters will appear. These characters can either be single characters (like ’j’, ’3’, or ’n’) or digraphs (which are pairs of characters like ’jo’, ’hn’, and ’23’). Each slot could either be a single character (which takes up one slot) or a digraph (which also takes up one slot, even though it’s made up of two characters). When a digraph is chosen to fill a slot, although a digraph contains two characters, it will be counted as if it is one character.

According to the example above, the selected characters are "o", "n", "e", "1", "2", "3", "4", "5", "6", and "j", and the list of digraphs is "jo", "oh", and "23".

Suppose a generated CAPTCHA would contain six characters and characters and digraphs are randomly selected from CAPTCHA Key database. Assume that the character "n","1","4","j", "hn", and "12" are randomly selected from CAPTCHA Key database. Then, the generated CAPTCHA could be “n12j14hn”.

For the above example, the generated CAPTCHA includes four single characters and two digraphs; therefore, one CAPTCHA should contain only six characters as expected, but finally, it consists of eight characters according to the two digraphs applied. In such a case, the user must solve six time slots while typing the CAPTCHA because each individual character and digraph contains keystroke events. Moreover, the selection of these characters is entirely random, which is a critical feature for security. By avoiding any predictable patterns, each CAPTCHA is made unique, which substantially reducces the chances of automated systems or unauthorized users being able to solve or replicate the CAPTCHA.

In this process, the security of the generated CAPTCHA is significantly enhanced through the principle of randomness. The system selects characters randomly, ensuring that there is no discernible pattern in the CAPTCHA challenges. This randomness is key to the uniqueness of each CAPTCHA, making it extremely difficult for unauthorized entities to predict or replicate the CAPTCHA values.

The probability for predicting the generated CAPTCHA with the five correct keystroke values can be calculated as follows.

Let *p*_*dw*_ be probability to press the keystroke with exact dwell time, Dw.
Let *p*_*In*_ be probability to press the keystroke with exact interval time, In.
Let *p*_*La*_ be probability to press the keystroke with exact latency time, La.
Let *p*_*Fl*_ be probability to press the keystroke with exact flight time, Fl.
Let *p_Up_* be probability to press the keystroke with exact up−to−up time, Up
Let $p_{dw}=p_{In}=p_{La}=p_{Fl}=p_{Up}\leq 0.5$.
*p*_*dw*_ occurs when a character is selected.
*p*_*In*_,*p*_*La*_,*p*_*Fl*_, *and p*_*Up*_ occur only when a digraph is selected.
Let *N* be all possible characters that can be generated as a CAPTCHA.
Let *n* be number of true characters of generated CAPTCHA.
Let *n*_*d*_ be number of digraphs in the generated CAPTCHA.
Let *n*_*s*_ be number of single characters in the generated CAPTCHA.
Let *p*(*n*) be probability to break the generated CAPTCHA.

Since *N* is all possible characters that can be generated as a CAPTCHA, then *N* could be one of 26 English characters and 10 numbers.


$$n=n_{d}+n_{s}$$

p(n)=1(Nn)×(p(nd)×p(ns))

p(n)=1(Nn)×((pInnd×pLand×pFlnd×pUpnd)×pdwns)

p(n)≤1(Nn)×(0.54nd+ns) (3)

Thus, if the generated CAPTCHA is “n12j14hn”, *n* = 8, *n*_*d*_ = 2, and *n*_*s*_ = 4, then *p*(*n*)≤8.07×10^−12^ and is close to zero.

Thus, from Eq (3), if the generated CAPTCHA contains many digraphs or long single characters, the probability of breaking this CAPTCHA is to converge into zero. Fig 5 shows examples of text-based CAPTCHAs from the John Doe profile under the six-slot structure.

**Fig 7:**
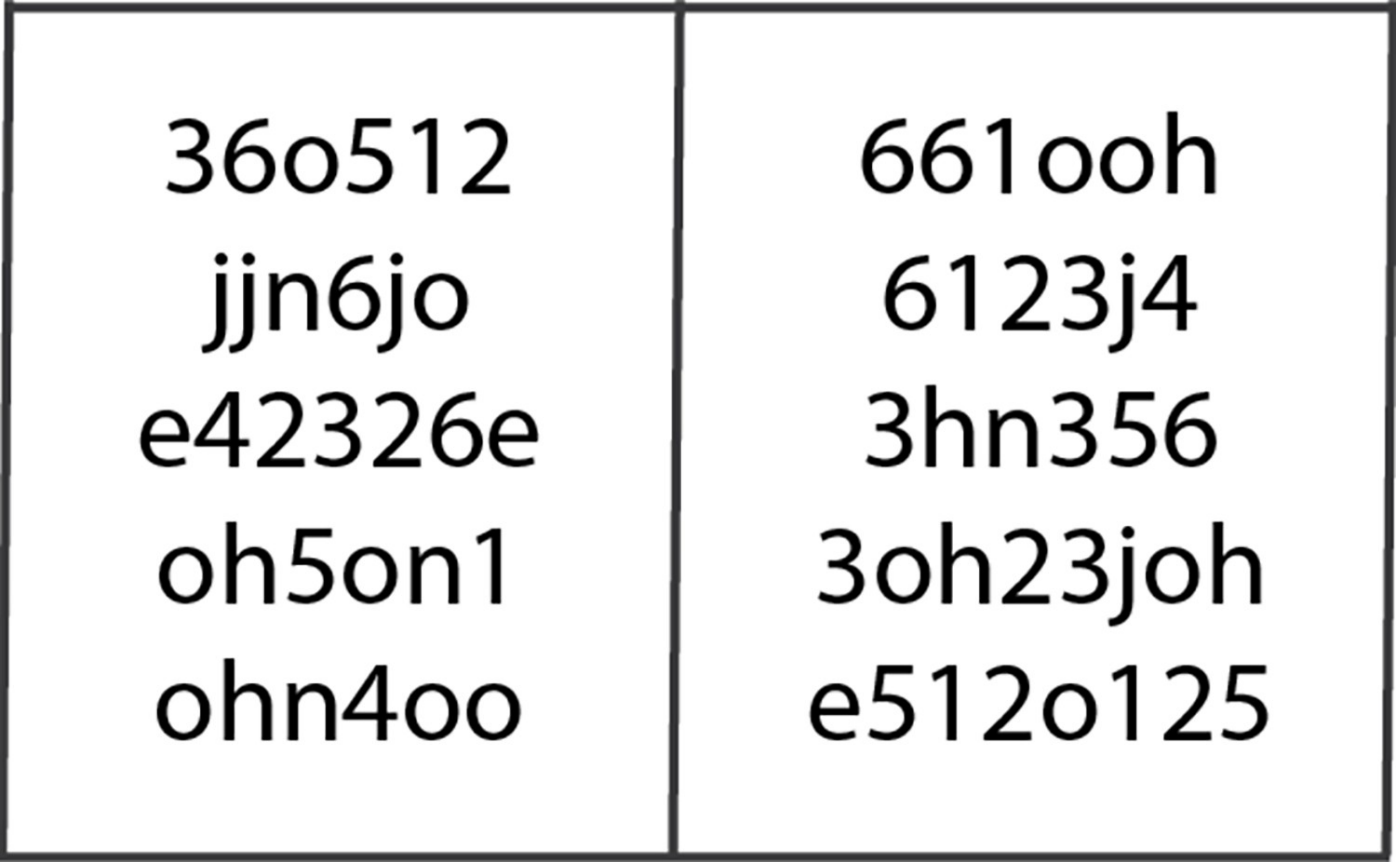
Examples of text-based CAPTCHAs created by the CAPTCHA Generator from John Doe profile.

Based on the examples in Fig 7, although the generated CAPTCHAs are based on a six-slot structure, the actual lengths of these generated CAPTCHAs may be longer than six characters per CAPTCHA. This is because the CAPTCHAs are composed by randomly selecting from a pool of frequently used single characters and digraphs a pair of characters treated as a single unit. Users are required to type all visible characters to solve the CAPTCHA, acknowledging the digraph as two separate characters for input purposes, while the system internally counts the digraph as a single slot. This method ensures the security of the CAPTCHA by avoiding predictability and ease of use for individuals familiar with their own frequently typed characters and character pairs.

#### 4.3.3 CAPTCHA input module

The user inputs the CAPTCHA challenge. The system captures and analyzes the real-time keystroke dynamics during this process. Following the generation of a Text-based CAPTCHA, the user is prompted to input this CAPTCHA on the CAPTCHA Input screen. This initiates the feature validation process.

#### 4.3.4 Feature validation module

The system analyzes the CAPTCHA data, focusing on the matching of input strings with the keystroke dynamics stored in the ’CAPTCHA Key database. The verification process relies on both behavioral biometrics data and input strings. The keystroke checking will be compared individually based on the typing of single characters or digraphs. The following algorithm is responsible for the validation process that indicates the authenticated user or an impostor.

**Fig 8:**
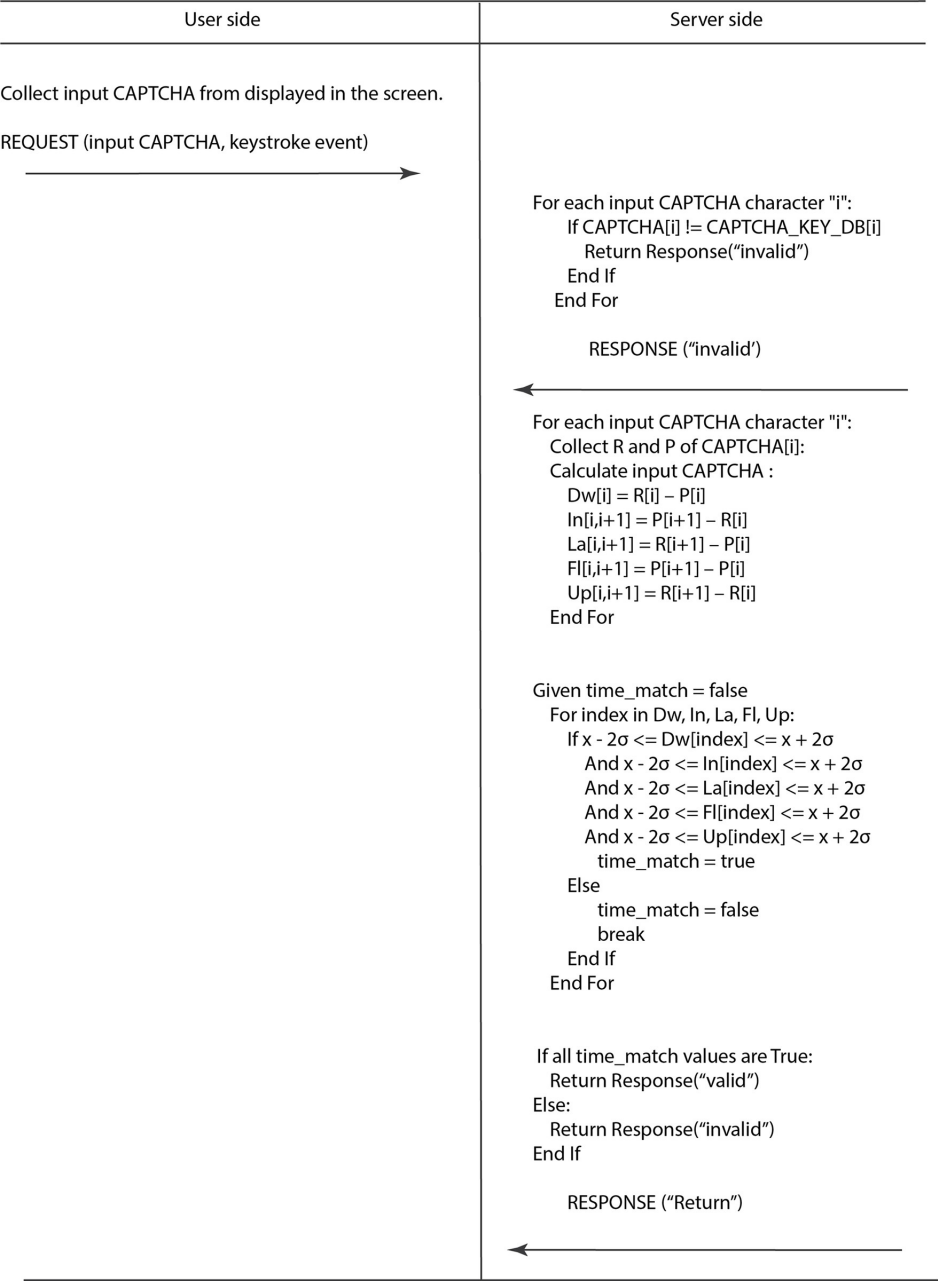
The feature validation algorithm.

The Fig 8 provides s a validation process between a user and a server, specifically focusing on CAPTCHA verification through keystroke dynamics. Initially, the user is presented with a CAPTCHA challenge on-screen and begins to input the solution. As the user types, each keystroke and corresponding event is collected. This information, along with the user’s identifier, is then sent to the server as a request for validation.

Initially, the user is presented with a CAPTCHA challenge on-screen and proceeds to input the required characters, capturing both the characters themselves (input_key_i) and the associated keystroke events (input_key_event_i).

Following data collection, the user sends this information to the server, which then commences a two-fold validation process. The server first verifies the accuracy of the CAPTCHA characters entered by the user against a randomly generated CAPTCHA from the set of values in the CAPTCHA Key Database (CAPTCHA_KEY_DB). If the characters entered do not match the generated CAPTCHA, the server immediately deems the attempt “invalid” and returns an “invalid” response.

If the user’s input matches the generated CAPTCHA, the server then analyzes the timing of the keystrokes. It calculates specific metrics for each keystroke, such as the Dwell Time (Dw), Interval Time (In), Latency Time (La), Flight Time (Fl), and Up-to-Up Time (Up), using the press (P) and release (R) points of each keystroke. Each of these calculated times is then checked against a statistical range defined by the mean ($\bar{x}$) and standard deviation (σ) of expected input times—essentially verifying that the timing of each keystroke falls within two standard deviations of the mean ($\bar{x}$ - 2σ ≤ input_time_value ≤ $\bar{x}$ + 2σ).

The server sets a time_match flag to false if any of the timing metrics fall outside of this acceptable range ($\bar{x}$ - 2σ ≤ input_time_value ≤ $\bar{x}$ + 2σ). If all metrics are within the range, the time_match remains “true”, indicating a human-like entry pattern. The final outcome of the server’s validation process hinges on the time_match flag; if all are “true”, a “valid” response is sent to the user. Conversely, if any timing metric fails the validation, an “invalid” response is issued.

The server’s response, be it “valid” or “invalid,” is then transmitted back to the user, completing the CAPTCHA validation sequence. This meticulous process ensures the integrity of the CAPTCHA challenge by affirming not only the correctness of the characters but also the authenticity of the entry pattern, effectively distinguishing human users from automated systems.

Based on the details for John Doe, the required CAPTCHA sequence is “n12j14hn”. This sequence is comprised of the key elements “n”, “1”, “2”, “4”, “j”, “hn”, and “12”. The corresponding keystroke dynamics are shown in Table 3.

Based on the values in Table 4, which presents sample timing values for keystrokes associated with the characters of the CAPTCHA sequence “n12j14hn”. The table includes timing data of dwell time for four distinct characters: “n”, “1”, “4”, and “j”, as well as interval time, latency time, flight time, and up to up time of sequences characters such as “hn” and “12”. The timing data consists of the mean time ($\bar{x}$) and standard deviation (σ) for each character or sequence (digraph) as recorded during the user’s enrollment phase. For example, the character “n” has an average ($\bar{x}$) input time of 91.840 milliseconds, with a natural variability (σ) of 12.599 milliseconds. Similarly, the character “1” has an average ($\bar{x}$) input time of 109.466 milliseconds with a variability (σ) of 12.872 milliseconds, and the character “4” has an average ($\bar{x}$) input time of 99.95 milliseconds with a variability of 22.647 milliseconds. Moreover, the character “j” in the table indicates a mean ($\bar{x}$) input time of 109.467 milliseconds and a standard deviation (σ) of 12.610 milliseconds.

**Table 4:** The sample keystroke timing values for all components of the CAPTCHA "n12j14hn" and the range for acceptable timing.

Key event	n ($\bar{x},sd$)	1 ($\bar{x},sd$)	4 ($\bar{x},sd$)	j ($\bar{x},sd$)	hn ($\bar{x},sd$)	12 ($\bar{x},sd$)
Dw	82 (91.840,12.599)	120 (109.466,12.872)	102 (99.95,22.647)	109 (109.467,12.610)	-	-
In	-	-	-	-	221 (217.063, 24.834)	211 (157.642, 21.095)
La	-	-	-	-	384 (398.5, 24.979)	419 (387.214, 37.369)
Fl	-	-	-	-	307 (314.313, 24.149)	331 (287, 22.281)
Up	-	-	-	-	398 (301.25, 23.507)	299 (257.857, 33.475)

Additionally, Table 4 also contains the time values during the CAPTCHA test. For the character “n”, the user entered the character “n” in 82 milliseconds, while for the character “1”, the user typed the character “1” in 120 milliseconds. Then, for the character “4”, the user inputs “4” in 102 milliseconds; for the character “j”, the current input time from the user is 109 milliseconds.

Besides, Table 4 also presents the information on digraphs that are used to generate a CAPTCHA challenge, “hn” and “12”. For the sequences “hn” and “12”, the table lists various timing values: interval time (In), latency time (La), flight time (Fl), and up to up time (Up). For the ’hn’ sequence, the interval time measures the duration from initiating the press of the “h” key to pressing the “n” key, with an average of 217.063 milliseconds and a standard deviation of 24.834 milliseconds. In addition, the latency time—the time from releasing “h” to pressing “n”—has a mean of 398.5 milliseconds and a standard deviation of 24.979 milliseconds. Then, the measurement of the flight time average for “hn”, representing the transition from “h” to “n”, is 314.313 milliseconds with a standard deviation of 24.149 milliseconds. Finally, the up-to-up time, the duration from releasing “h” to releasing “n”, has averaged 301.25 milliseconds with a standard deviation of 23.507 milliseconds.

As described in single characters, the results in Table 4 for the CAPTCHA test show that the interval time is logged at 221 milliseconds, the latency time for ’hn’ is 384 milliseconds, the flight time can be counted as 307 milliseconds, and the up-to-up time is 398 milliseconds for the user.

Using the values in Table 4 in the validation process demonstrated in Table 5 as follows:

**Table 5:** The validation process of a random generated CAPTCHA "n12j14hn".

Character	Key event	Input time value	$\bar{x}-2\sigma$	$\bar{x}+2\sigma$	output
n	Dw	82	66.64109	117.04007	Valid
1	Dw	120	88.73885	140.22667	Valid
4	Dw	102	54.65576	152.56452	Valid
j	Dw	109	84.24635	160.22458	Valid
12	In	221	167.3944	266.730565	Valid
12	La	384	348.5400	448.459984	Valid
12	FL	307	266.014985	362.610015	Valid
12	Up	398	254.235109	348.264891	Valid
hn	In	211	115.45203	199.833685	Valid
Hn	La	419	312.475032	461.953539	Valid
Hn	Fl	331	242.437166	331.562834	Valid
hn	Up	299	190.906612	324.807674	Valid

Table 5 provides a validation framework for the ProCAPTCHA system when comparing the times of keystrokes and the predetermined valid time domains for each character or sequence in the CAPTCHA Key database. If a true input time value is in the range of, then the output result is “Valid”; otherwise, it is “Invalid”.

Referring to the rule in the previous paragraph, each keystroke or sequence listed in Table 5 falls within the respective valid timing ranges, which means they all receive a "Valid" output. The system uses these timings to authenticate the user’s input; if all the inputs are within the valid ranges, the overall CAPTCHA entry is considered valid. Conversely, if any input fell outside the valid ranges, it would likely be flagged as invalid, potentially indicating non-authentic or suspicious entry behavior.

#### 4.3.5 Decision Presentation Module

The Decision Presentation Module is a critical component of the ProCAPTCHA system, serving as the final stage where the outcome of the user’s login attempt is communicated. This module operates on a straightforward principle: it presents the result of the verification process directly to the user, indicating whether their attempt was successful or not.

After passing the validation test from the Feature Validation module, if the input string matches with the randomly generated CAPTCHA and every comparison result is “Valid”, then the presented message on the screen is “success”. This signals that the login process has been completed and access is granted.

On the other hand, if there is a mismatch in the CAPTCHA or a deviation in the keystroke pattern from the user’s profile, the system detects an anomaly. This triggers a "fail" screen, notifying the user that the login attempt has been unsuccessful. This serves as a direct alert that the verification process has identified irregularities, and access is consequently denied, safeguarding against potential unauthorized access.

In essence, the Decision Presentation Module’s role is to clearly communicate the result of the login attempt to the user, ensuring they are immediately aware of their authentication status—whether they have been authenticated as a user or flagged as an intruder.

## 5. Security analysis

In the evolving realm of online security, CAPTCHA systems stand as critical gatekeepers, protecting digital platforms from automated attacks and unauthorized access. The study’s exploration of ProCAPTCHA marks a significant stride in this domain, introducing a novel system that intertwines user-specific biometric data with CAPTCHA technology. This security analysis aims to dissect and evaluate the robustness of ProCAPTCHA, delving into its efficacy in deterring both human and automated threats.

Given the escalating sophistication of cyber threats, particularly in the realm of identity verification and automated bot attacks, the introduction of ProCAPTCHA presents an intriguing solution. By leveraging unique user behavioral biometrics, specifically keystroke dynamics, in combination with personalized CAPTCHA challenges, ProCAPTCHA proposes an innovative approach to secure user authentication.

The analysis is rooted in a detailed examination of ProCAPTCHA’s performance under various attack scenarios, including both human intrusions and automated bot attacks. It delves into the system’s resilience against traditional attack vectors like brute force and its adeptness in handling sophisticated bot attacks that mimic human interaction patterns. Additionally, it contemplates the implications of using personalized data in CAPTCHA generation, discussing the balance between enhanced security and potential challenges in user experience and data privacy.

### 5.1 Probability of breaking the ProCAPTCHA

The probability calculations for correctly guessing the ProCAPTCHA play a pivotal role in its security efficacy. The following section draws examples of finding probability in breaking a ProCAPTCHA.

#### 5.1.1 Probability of breaking the ProCAPTCHA

*Low probability of correct guesses*. The security strength of ProCAPTCHA lies significantly in the mathematical improbability of correctly guessing ProCAPTCHA. Based on the probability model, Formula 1, stated in Section 4.3.2, the probabilities of breaking ProCAPTCHA’s examples in Table 6 can be computed as follows:

**Table 6:** The estimated probability in breaking individual ProCAPTCHA.

ProCAPTCHA	Number of Single and Digraph	Probability (*p*(*n*))
n12j14hn	(4, 2)	8.07×10^−12^
661ooh	(6,0)	8.022×10^−9^
jjn6jo	(6,0)	8.022×10^−9^
e512o12oh	(3,3)	3.24×10^−13^

Based on the results in Table 6, the probability formula considers the number of possible character permutations and the intricacies of typing dynamics, such as dwell time and flight time, for each character and digraph. In this case, the combination of 26 English characters and ten numerals leads to an exceedingly high number of possible permutations, making a correct guess extremely unlikely. Since there is only one ProCAPTCHA selected from this set of possibilities, the probability of correctly guessing the ProCAPTCHA is approximately close to zero for every word of ProCAPTCHA. The possibility of successfully guessing and attacking this ProCAPTCHA is quite low. A random generator can be beneficial for security purposes. Furthermore, the security of the ProCAPTCHA is determined by the number of digits employed.

*Statistical analysis*. The probability calculation integrates statistical concepts by considering the average ($\bar{x}$) and variability (*σ*) of keystroke timings for each character. This approach ensures that the system doesn’t just rely on the correct sequence of characters but also on the specific timing patterns associated with them, unique to each user. The range within two standard deviations (95% confidence interval) forms the basis for determining whether an input pattern matches the expected user behavior. This statistical rigor in the validation process greatly reduces the likelihood of an unauthorized user successfully replicating the necessary keystroke dynamics.

*Impact of randomness*. In each CAPTCHA challenge, the probability of correctly guessing the characters and digraphs is greatly reduced by their random selection, according to the additional use of typing timeframes. This application of randomness, a fundamental concept where outcomes lack predictability and discernible patterns, ensures the uniqueness and unpredictability of each ProCAPTCHA, complicating the efforts of potential attackers who cannot depend on static or predictable patterns.

Employing computational randomness for the selection of characters and digraphs transforms each CAPTCHA challenge into a unique realization of randomness. This approach aligns with the principles of randomness theory [43], where each outcome in a sequence is statistically independent and identically distributed, much like individual events in a random process. In the context of ProCAPTCHA generation, this translates to each challenge being an independent instance of such a process, with the chosen characters and digraphs representing the random variables.

This theory, applied to ProCAPTCHA generation, mirrors the inherent unpredictability of random processes akin to the evolution of a noisy signal over time. The randomness inherent in ProCAPTCHAs, thus, significantly lowers the likelihood of accurate predictions. The unpredictable selection of characters and digraphs fosters a high entropy environment within each ProCAPTCHA instance. This not only ensures the distinctiveness of each challenge but also negates the effectiveness of pattern recognition tactics. Consequently, the security of the ProCAPTCHA system is enhanced, making it a formidable barrier against intrusion and automated attacks, which is in line with the key principles of randomness theory.

#### 5.1.2 Complexity with digraphs

*Enhanced security with digraphs*. The integration of digraphs, which are pairs of characters treated as a single unit, significantly complicates the CAPTCHA challenges. Unlike single characters, digraphs incorporate an additional dimension of complexity as they require the correct sequence of two characters, along with their unique typing dynamics, to be inputted correctly. As shown in Table 6, a high frequency of digraphs causes a lower probability of success in breaking the ProCAPTCHA word than using only single characters.

Typing Dynamics of Digraphs: When a user types a digraph, the system measures not just the individual dwell times of each character but also the interval, latency, flight, and up-to-up times between the two characters. These time intervals represent the duration between releasing one character and pressing the next and vice versa. Such detailed measurement of the interaction between characters in a digraph makes it exponentially more difficult for an unauthorized user or a bot to replicate the exact typing pattern.

*User familiarity and security*. The use of digraphs from a user’s frequently typed data (like names, phone numbers, or email addresses) implies that while the ProCAPTCHA is inherently complex, it remains user-friendly for the legitimate user. This personalized approach maintains security without compromising user convenience.

The low probability of correctly guessing a ProCAPTCHA challenge, bolstered by the complexity introduced through the use of digraphs, demonstrates the system’s robustness against unauthorized access attempts. This dual-layered approach–combining statistical probability with behavioral biometrics–makes ProCAPTCHA a formidable defense in the realm of online security.

### 5.2 Effectiveness against automated attacks

#### 5.2.1 Virtual ciphertext attack

When assessing the efficacy of ProCAPTCHA in mitigating automated attacks, particularly those initiated by bots, it becomes apparent that the system functions as a resilient defense mechanism. This evaluation centers on the system’s resilience against such assaults, specifically highlighting its countermeasures against a virtual ciphertext attack, which emulates ciphertext attacks. The virtual ciphertext assault entails the assailant obtaining the ProCAPTCHA word without being cognizant of the character-specific significant time values. Those time values must be identified by an automated attack program in order to obtain access to the system. Consequently, every substantial time value functions as a private key; for every character or digraph of the ProCAPTCHA that the algorithm is obligated to acquire, the ProCAPTCHA will be compromised.

**Fig 9:**
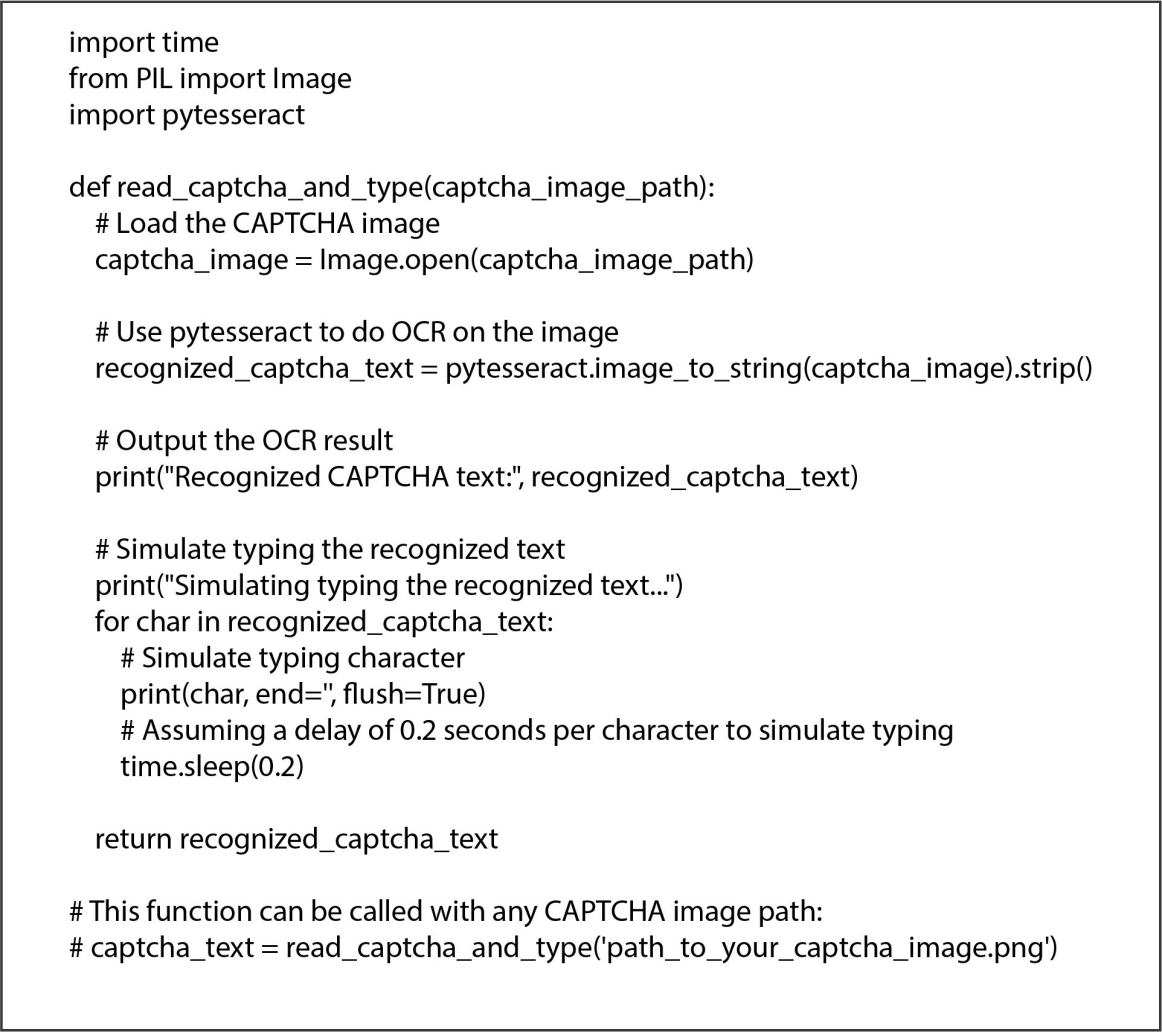
The pseudocode to solve ProCAPTCHA are as the follow.

An automated attacks program simulator was utilized to assess ProCAPTCHA’s performance, as illustrated in Fig 9. The simulator program leverages several components, including image processing using the pytesseract library for the Optical Character Recognition (OCR). In this research, an attacker knows a ProCAPTCHA word while trying to gain access to the system. Thus, an algorithm to perform the ciphertext attack is shown below.

Basically, the attacker uses OCR to solve the presented CAPTCHA; in such cases, some OCR misinterpretations were observed during the attacking simulation process. For instance, in the second CAPTCHA “661ooh”, the letter “o” was misinterpreted as “0”, and in the fourth CAPTCHA “e512o125”, a special character “€” was read instead of “e”. These misinterpretations further enhance ProCAPTCHA’s security by introducing variability and complexity for bots trying to bypass the CAPTCHA, thereby preventing automated access to the system.

Besides using the OCR, the impostors use a simple technique called Brute Force to break a CAPTCHA or any key value. Table 7 presents the results of the simulations, indicating the estimated time required for the Brute Force simulator bot to successfully solve individual ProCAPTCHA series. However, this simulator bot cannot simulate the keystroke time values that match the owner’s time values. Thus, the bot cannot pass the validation process, although the ProCAPTCHA characters are completely matched.

**Table 7:** The estimated time for each individual CAPTCHA series by simulation bot.

CAPTCHA test	Character	Time to solve by bots	Access to the system
661ooh	6,6,1,o,o,h	2.45 seconds	Failed
jjn6jo	J,j,n,6,j,o	2.45 seconds	Failed
n12j14hn	n,12,j,1,4,hn	2.85 seconds	Failed
e512o12oh	e,5,12,o,12,o,h	3.05 seconds	Failed

The effectiveness of the safeguard against bots and assailants provided by the assumption of time values and significant characters is illustrated through the execution of a simulation utilizing an automated time-delay attack program. This simulation program evaluates the robustness of ProCAPTCHA using the pytesseract, Phyton’s library for OCR. The results of this simulation demonstrated that despite a bot possessing knowledge of a ProCAPTCHA word and maintaining a consistent typing cadence of 0.2 seconds per character, it fails to accurately replicate the intricate timing of human typing, which differs between keys. Typing time intervals can be considered an additional security measure due to the fact that automated entries may be detected and thwarted by bots, which are unable to inherit typing.

Since the ProCAPTCHA system uses time values associated with each character or digraph (pair of characters) as a security feature, these time values act like “private keys”. Therefore, to successfully solve a ProCAPTCHA, an automated program (bot) would need to know whether the character is single or a part of a digraph and also the correct timing for either individual character or digraph. The assumption is that without this knowledges, the chance of a bot deciphering the ProCAPTCHA is extremely low.

#### 5.2.2 Advanced bot attacks

A time delay mechanism is utilized by the advanced malware attack to simulate human typing patterns as a type of emulator. The evaluation of the proposed system’s performance is conducted by counting the duration of time it takes for this program to circumvent the ProCAPTCHA. The bot is able to circumvent the ProCAPTCHA if the breaking time falls within the acceptance time interval of the authenticated user. If not, the protection is complete.

The advanced bot attacks are characterized by their ability to interpret the ProCAPTCHA text through high-level image processing, akin to what was observed with simpler bots. However, the critical enhancement in these bots lies in their typing simulation. Unlike basic bots that input recognized characters with consistent timing, advanced bots employ an algorithm that introduces random delays in typing, similar to the natural variances seen in human typing behavior. These delays are not uniform; they include shorter bursts of rapid typing, occasional longer pauses (possibly simulating moments of thought or error correction), and regular intervals of typing that closely mimic human interaction. Based on the experiment samples, the minimum value of typing time is 0.1 seconds, and the maximum value is 0.7 seconds. Thus, the range of random delay is between [0.1, 0.7].

In this advanced scenario, each significant time value used in typing the ProCAPTCHA characters still functions as a private key. However, the challenge is linearly increased based on the number of digraphs in the ProCAPTCHA text, as the bot must not only accurately decipher the text but also replicate the nuanced timing associated with human typing. This complexity adds a new layer to ProCAPTCHA’s security, as the system not only has to validate the correctness of the entered text but also analyze the typing pattern for signs of automated input.

An enhanced simulation program was deployed for this evaluation, incorporating elements of human-like typing patterns along with OCR capabilities. As depicted in Fig 8, this simulator demonstrates a higher level of sophistication in attempting to breach the ProCAPTCHA system. The key question under investigation is whether the ProCAPTCHA can distinguish between these advanced bots and a genuine human users, particularly when bots are designed to closely imitate human typing rhythms and patterns.

**Fig 10:**
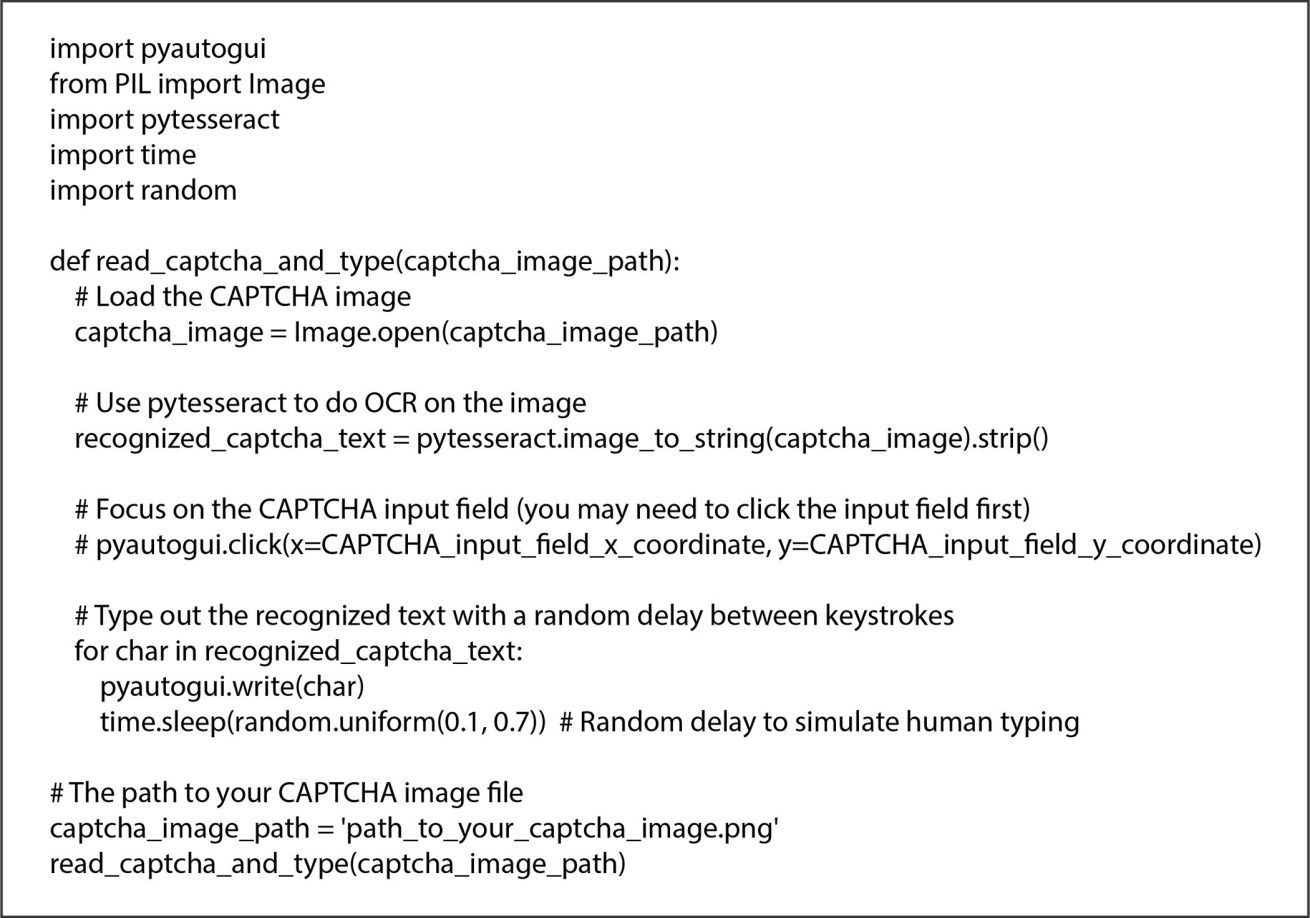
A script to solve ProCAPTCHA with human-like typing simulation.

From Fig 10, The program is designed to replicate human-like typing behavior when interacting with CAPTCHA images. To achieve this, a crucial element: a random time delay is introduced between each keystroke. This random delay serves to mimic the natural variability in human typing rhythms. It is generated using a random function.uniform(0.1, 0.7), which produces a random floating-point value between 0.1 and 0.7 seconds. As a result, the program types each character of the CAPTCHA text with unpredictable pauses and bursts, simulating the authentic typing pattern of a human. This range of delays, sometimes short and at other times longer, adds an element of unpredictability to the typing speed. The overall typing speed of the program depends on the length of the CAPTCHA text, as the cumulative effect of these random delays influences the time it takes to complete the typing process. This variability in typing rhythm is a critical aspect of the program’s ability to mimic human typing and makes it challenging for automated systems to distinguish it from genuine human input.

As mentioned previously the typing time is totally related to the number of digraphs in a ProCHAPCHA is in the linear form, Table 8 provides the times to solve the ProCAPTCHAs range from 3.87 seconds to 6.02 seconds, suggesting that the Advanced Brute Force bot took longer to solve more complex ProCAPTCHAs. The table highlights the advanced nature of the bot’s simulation, which attempts to mimic human typing patterns by introducing random delays between keystrokes. Despite this sophistication, the bot was unable to solve any of the ProCAPTCHA tests within a time frame or with the accuracy that would be deemed human-like, resulting in a "Failed" status across all tests.

**Table 8:** The estimated time for each individual CAPTCHA series by advance simulation bot.

CAPTCHA test	Character	Time to solve by Advanced bots	Access to the system
jjn6jo	J,j,n,6,j,o	3.87 seconds	Failed
p75609x	p,7,56,0,9,x	4.21 seconds	Failed
n12j14hn	n,12,j,1,4,hn	5.89 seconds	Failed
e512o12oh	e,5,12,o,12,oh	5.97 seconds	Failed
ndxa90p834	nd,xa,90,p,8,34	5.83 seconds	Failed
do34nomleex	do,34,n,om,le,ex	5.66 seconds	Failed
om6778lendam	Om,67,78,le,nd,am	6.02 seconds	Failed

These times are determined through a simulation that incorporates random delays between 0.1 and 0.7 seconds per character, closely emulating human typing speed. It’s important to note that in a real-world scenario, the actual solving times may vary due to factors such as human typing speed variations and the accuracy of Optical Character Recognition (OCR) software in correctly identifying the characters.

**Fig 11:**
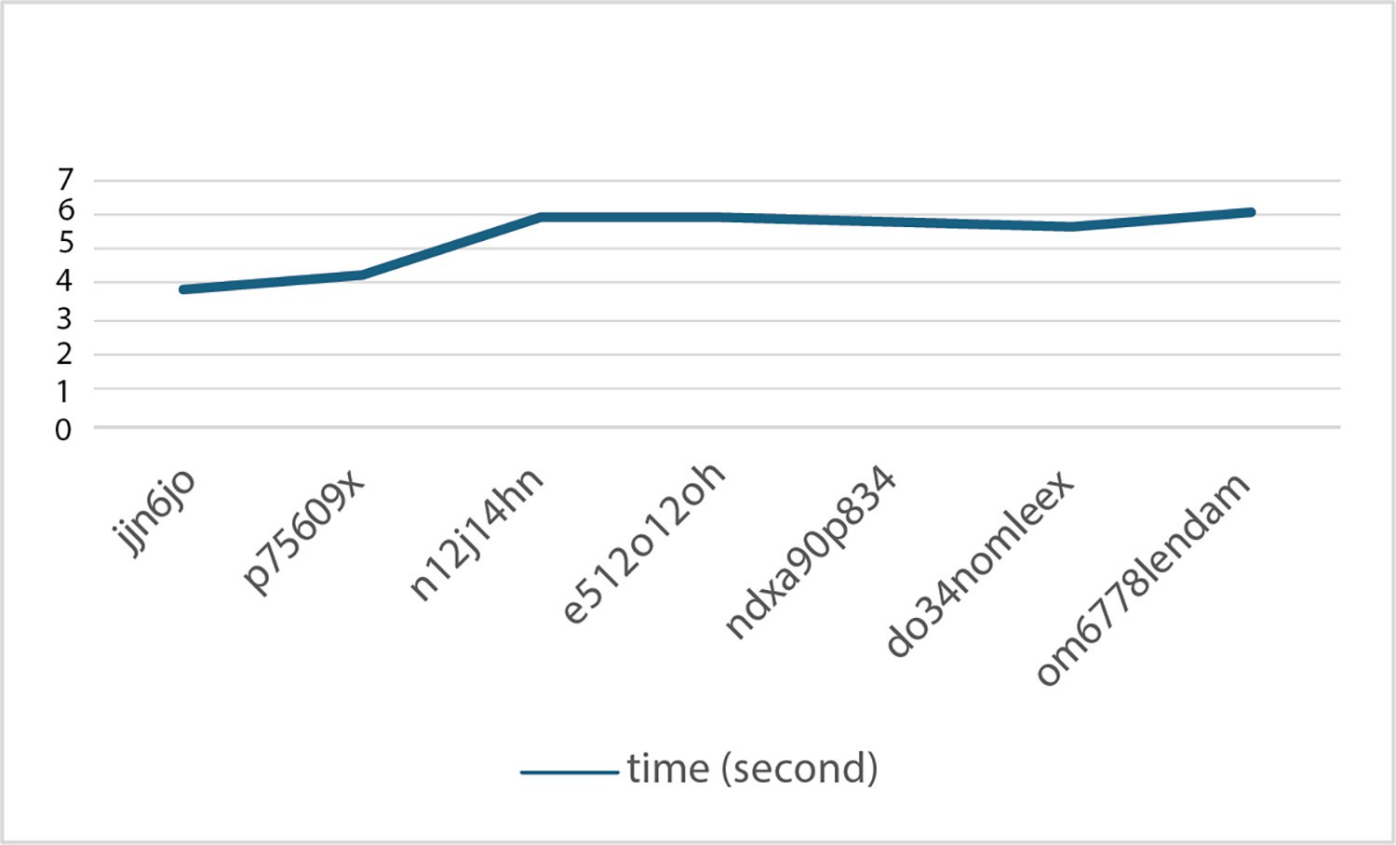
Graph of time taken to solve ProCAPTCHAs.

Below Table 8 is a line graph of Fig 11, which plots the ProCAPTCHA tests on the x-axis against the time taken to solve them (in seconds) on the y-axis. This visual representation helps quickly compare the time taken for each ProCAPTCHA test, showing a general trend where more complex ProCAPTCHA strings (possibly longer or with more complex character combinations) take longer to solve.

The typing time equation for an advanced bot that solves ProCAPTCHA takes into account individual reading times by OCR and individual typing delays for each character. That model is constructed using the following parameters:

- *t*_*ocr*(*i*)_: Time for optical character recognition to process the *i*^*th*^ character.
- *d*_*i*_: Individually randomized typing delay for the *i*^*th*^ character, introduced by the bot.
- *n*: Count of characters in the CAPTCHA challenge.

The total time *n* for a bot to decipher a CAPTCHA is then formulated as the cumulative sum of individual OCR processing times and typing delays:

$$T=\sum_{i=1}^{n}\left(t_{ocr(i)}+d_{i}\right)$$

Where, *d*_*i*_ denotes the randomized delay, which could range from 0.1 to 0.7 seconds, effectively simulating human typing idiosyncrasies.

In a scenario where a ProCAPTCHA comprises of 6 characters, no digraph, and assuming the values of *t*_*ocr*(*i*)_ and, *d*_*i*_ are known for each character, the total time *T* can be calculated as follows:

$$T=\sum_{i=1}^{6}\left(t_{ocr(i)}+d_{i}\right)$$

This equation provides the framework to quantify the efficiency of bots in solving ProCAPTCHA tests and to analyze the security threshold of the ProCAPTCHA system. However, it can be seen that the solving time value of bots might exceed real human-being times because $\sum_{i=1}^{6}t_{ocr(i)}$ is usually larger than a human’s sense.

So, this advanced scenario presents a challenge for ProCAPTCHA systems, which must now account for not just the accuracy of text recognition but also the pattern of typing, whether single or digraph, including keystroke time intervals. The advanced simulation demonstrated that using the ProCAPTCHA can increase the security assessment because by breaking a ProCAPTCHA, the offenders must first know about the existence of digraphs and their positions. Then, all characters must be guessed using the Brute Force method. Moreover, while typing each character, the related typing time values must be randomly predicted. All these processes must be corrected and matched with the true owner, ProCAPTCHA. As a consequence, the probability of breaking a ProCAPTCHA is close to zero.

#### 5.2.3 Evaluating the efficacy of ProCAPTCHA systems using machine learning classification model

**Fig 12:**
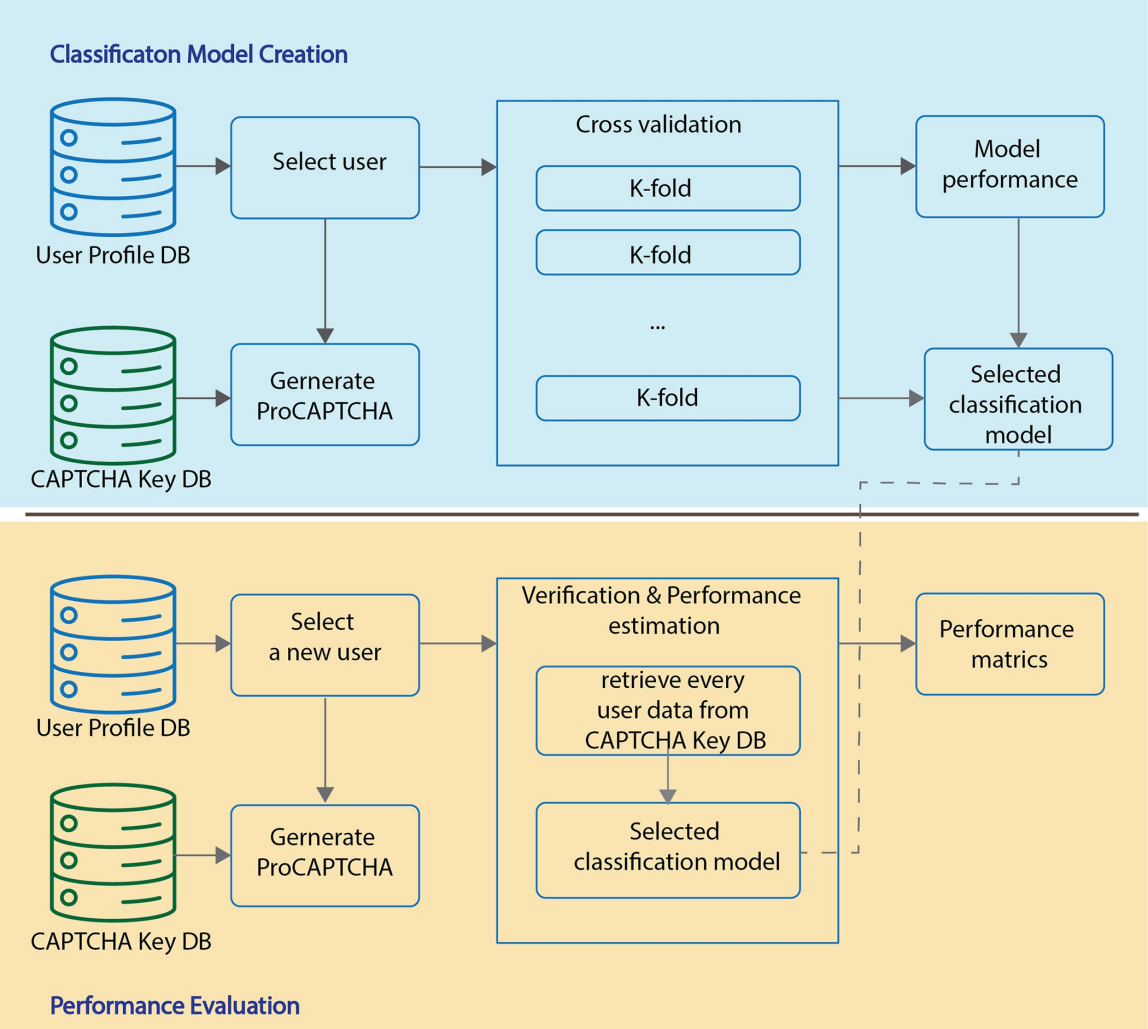
The evaluation process of ProCAPTCHA systems.

The evaluation process, illustrated in Fig 12, aims to determine the efficiency of a customized ProCAPTCHA system designed for each user. Allowing users to enter their own ProCAPTCHA and then simulating other users’ intrusion attempts to test the system’s security. Testing for the use of ProCAPTCHAs, including those with single characters and digraphs, is utilized to evaluate the model’s capability in handling different complexity levels and safeguarding against unauthorized access.

Fig 10 outlines a systematic method for assessing the ProCAPTCHA system through the use of machine learning algorithms. The framework examines the performance of three machine learning models: Decision Trees, Random Forests, and Gradient Boosting Trees. Initially, the process commences with the selection of an individual from a User Profile Database, for whom a customized ProCAPTCHA is generated based on the data available in the CAPTCHA Key Database, CAPTCHA Key DB. This personalized CAPTCHA generation suggests a tailored approach to user verification. Subsequently, the model undergoes a rigorous k-fold cross-validation process to ascertain its predictive accuracy, involving the partitioning of data into “k” subsets and utilizing each subset in rotation as a test set, while the others comprise the training set. Upon completion of the cross-validation, the most efficient classification model is selected based on its performance across these subsets. This phase also involves an in-depth analysis of how users interact with the ProCAPTCHA system, which helps the algorithms learn how to distinguish between legitimate use and fraudulent attempts.

The “Classification Model Creation” stage is vital in evaluating the models’ ability to provide security against unauthorized access while maintaining user convenience for legitimate users. After this initial testing, the algorithm that performs best, as indicated by metrics such as accuracy, precision, recall, and F-score, is selected to proceed to the next stage, “Performance Evaluation”. This strategic decision is taken to improve the overall efficacy, reliability, and security of the ProCAPTCHA system. Using the most effective model from the first phase, the system ensures strong protection against cyber threats and a user-friendly interface for genuine users.

In Phase 2, the “Performance Evaluation” stage, the chosen model is tested with a new user data and a range of ProCAPTCHA challenges. This intensive testing is crucial to evaluate the model’s resilience and adaptability thoroughly. This comprehensive evaluation is vital to ensure that the ProCAPTCHA system is sufficiently protected against various security threats. This stage is crucial in confirming the system’s ability to handle different scenarios effectively and maintain its reliability in protecting user data and access.

To assess the ProCAPTCHA system’s effectiveness, three primary performance metrics are employed: accuracy, precision, recall, and F-score. These metrics are key performance indicators of the system’s ability to accurately classify CAPTCHA challenges.

- Accuracy is the measure of the model’s overall correctness and is calculated as the ratio of correctly classified instances (both true positives and true negatives) to the total number of instances. The formula for accuracy is:

$$Accuracy=\frac{\sum \left(a+b\right)}{\sum \left(a+b+c+d\right)}$$

- Precision reflects the model’s exactness, representing the proportion of true positive instances among all instances that the model classified as positive. The precision formula is:

$$Precision=\frac{\sum a}{\sum \left(a+c\right)}$$

- Recall (or sensitivity) indicates the model’s completeness, measuring the proportion of actual positives that were correctly identified. The formula for recall is:

$$Recall=\frac{\sum a}{\sum \left(a+d\right)}$$

Where the variables represent:

*a*: True Positives—Instances correctly identified as positive
*b*: True Negatives—Instances correctly identified as negative
*c*: False Positives—Instances incorrectly identified as positive
*d*: False Negatives—Instances incorrectly identified as negative
  - The F-score is the harmonic mean of precision and recall, providing a single metric that balances both the model’s precision (its ability to identify only relevant instances) and its recall (its ability to identify all relevant instances). It’s especially useful in situations where the balance between precision and recall is important. For example, in ProCAPTCHA systems, the balance is needed to accurately distinguish between human users and intruders without wrongly denying access to real users or granting it to intruders.

These metrics are crucial for understanding the model’s performance in scenarios where the cost of false positives and negatives varies. Precision and recall are especially important in the context of CAPTCHA systems, where denying access to a legitimate user (false negative) or granting access to an intruder (false positive) can have significant implications.

#### 5.2.4 The efficacy of ProCAPTCHA systems

From evaluating the efficacy of ProCAPTCHA systems using Gradient Boosting Trees (GBT), Decision Trees, and Random Forests through cross-validation involves a comprehensive and structured approach to ensure that the chosen model performs optimally in the real-world scenarios. This process starts with data preparation, where the ProCAPTCHA dataset is meticulously preprocessed to extract relevant features, such as converting images into grayscale and normalizing pixel values. The dataset is then divided into *k* equal segments or folds, commonly opting for a *k* = 10 setup to facilitate a detailed cross-validation process.

The Cross Validation Operator implements an advanced strategy for assessing machine learning models’ efficiency, utilizing a structured method that splits the input data into *k* equal parts. This technique ensures that each segment alternately acts as a test dataset in one round and as part of the training dataset in the other rounds through k cycles of distinct training and testing phases. Such a detailed procedure guarantees the utilization of each data point for both learning and validation purposes; thereby, this detailed procedure offers an in-depth evaluation of the model’s accuracy and its capability to adapt to new, unseen data. By calculating and then averaging the performance metrics from each cycle, the operator delivers a solid estimate of the model’s performance, simultaneously checking for overfitting, which affirms the model’s dependability for real-world application.

During the cross-validation stage, each model is subjected to a thorough scrutiny—within each of the *k* rounds, a unique fold is selected as the validation set, and the rest are amalgamated as the training set. This allows for the training and evaluation of each model across the entirety of the dataset, ensuring an all-encompassing review. Metrics such as accuracy, precision, recall, and F-measure are determined for each fold and averaged out to provide a comprehensive perspective on each model’s effectiveness.

Once the cross-validation is complete, the outcomes are compiled, enabling a straightforward comparison among the models based on the average values of the assessed metrics. This comparison goes beyond mere average performance to examine the consistency of each model across various subsets of data, as shown by the standard deviation of these scores. This critical analysis aids in discerning the subtle differences between models, which is particularly important in situations where striking the right balance between security measures and user convenience is essential. For example, in ProCAPTCHA systems, a slightly less accurate model that offers higher precision could be more desirable to prevent the unwarranted blocking of legitimate users (false positives) rather than inadvertently allowing bots (false negatives).

The decision on the final model to be adopted is made by aligning with the ProCAPTCHA system’s specific needs, taking into account the balance required between deterring unauthorized access and minimizing inconvenience to genuine users, alongside considerations of the model’s complexity and the computational resources available. Employing cross-validation in the evaluative phase ensures that the selection is based on a comprehensive and reflective understanding of the model’s capability to offer robust protection against unauthorized access while ensuring a smooth user experience.

The evaluation of the ProCAPTCHA system involves assessing its effectiveness against a spectrum of ProCAPTCHA challenges using machine learning algorithms like Gradient Boosting Trees (GBTs), Decision Trees (DTs), and Random Forests (RFs), applied through a cross-validation methodology. The challenges are stratified into series based on their complexity:

ProCAPTCHA Series 1: Features single characters exclusively, such as k, m, 7, p, 9, and o.
ProCAPTCHA Series 2: Introduces a single digraph, ee, amidst single characters like o, e, 9, d, and m.
ProCAPTCHA Series 3: Incorporates two digraphs, ak and kd, alongside single characters a, m, 7, and 0.
ProCAPTCHA Series 4: Contains three digraphs, ak, de, and kd, with the rest being single characters e, o, and m.
ProCAPTCHA Series 5: Increases complexity with four digraphs, kd, ak, de, and ee, complemented by single characters 9 and 0.
ProCAPTCHA Series 6: Presents five digraphs, ak, de, ee, kd, and pa, with only one single character, o.
ProCAPTCHA Series 7: Maximizes difficulty with all slots filled by digraphs: pa, ee, kd, ak, de, and ak.

These series are designed to test the adaptability of the machine learning algorithms powering the ProCAPTCHA system, progressively increasing the challenge to assess how well the system can handle varying levels of complexity.

In the evaluation outlined earlier, three distinct machine learning algorithms—Gradient Boosting Trees (GBTs), Decision Trees (DTs), and Random Forests (RFs)—were utilized to gauge the effectiveness of a sophisticated ProCAPTCHA system. The system’s performance was rigorously tested across a range of ProCAPTCHA challenges, each designed to assess the algorithms’ proficiency in distinguishing legitimate user entries from potential automated attempts. The results of this comprehensive testing are tabulated in Table 9, as referenced in Fig 10.

**Table 9:** Performance metrics of the ProCAPTCHA system.

CAPTCHA	GBTs				DTs				RFs			
Series	accuracy	recall	precision	f-measure	accuracy	recall	precision	f-measure	accuracy	recall	precision	f-measure
1	98.95	96.8	93.75	96.17	94.68	98.78	97.91	96.69	96.04	91.39	90.32	78.87
2	98.89	97.65	94.07	96.21	97.05	99.21	99.9	98.36	94.43	69.57	95	78.88
3	99.13	98.34	94.68	96.32	96.67	97.65	98.35	98.67	95.65	96.65	93	84.78
4	98.45	97.49	95.37	97.83	98.78	97.37	96.98	97.67	96.75	93.29	94	89.52
5	98.79	96.36	96.45	97.57	96.06	96.85	76.98	89.57	97.57	96.45	95.48	86.54
6	98.56	97.98	97.45	96.64	98.46	86.89	97.35	89.46	97.64	93.67	89.9	79.89
7	98.79	97.56	95.69	96.54	96.47	98.54	98.67	97.52	96.67	92.87	91.78	79.64

These results capture the algorithms’ accuracy, recall, precision, and F-measure across seven distinct series of ProCAPTCHA challenges, each with increasing complexity due to the incorporation of digraphs—pairs of characters. The metrics in Table 9 reflect how well each algorithm was able to correctly identify and process the ProCAPTCHAs presented, providing a clear indication of their respective strengths and potential areas for improvement. The data shows that the GBTs maintained remarkable consistency and high performance throughout the series. As the same time the DTs excelled particularly in simpler challenges but faced some difficulties with more complex series. The RFs, on the other hand, showed a generally robust performance but with certain fluctuations that suggest a sensitivity to specific complexities in the ProCAPTCHA configurations.

The performance is measured using four metrics: accuracy, recall, precision, and the F-measure, as show in the results from Table 9. GBTs exhibit consistently high performance across all ProCAPTCHA series, maintaining accuracy rates above 98% and demonstrating a strong balance between recall and precision, as reflected in their F-measure scores. This indicates a robust ability to handle various ProCAPTCHA complexities without significant drops in performance.

Decision Trees show remarkable precision, particularly in the second ProCAPTCHA series, where they achieve near-perfect scores, suggesting a high specificity in simpler CAPTCHA challenges. However, there is a noticeable decline in precision in the fifth series, indicating potential difficulties with more complex CAPTCHAs that incorporate multiple digraphs. Despite this, DTs maintain high accuracy and F-measure scores overall, suggesting a general effectiveness in ProCAPTCHA recognition.

Random Forests, while displaying relatively high accuracy with the lowest at 94.43% and peaking at 97.64%, show some variability and lower recall and F-measure scores in certain series, specifically the second and sixth. This might point to challenges in handling ProCAPTCHAs with a specific arrangement of digraphs and could signal areas where model tuning could be beneficial.

In summary, while each algorithm has demonstrated proficiency in CAPTCHA recognition, GBTs stand out for their consistent performance across varying levels of complexity. Decision Trees, while effective overall, may require adjustments when confronting ProCAPTCHAs with higher digraph content. Random Forests, despite occasional dips in certain metrics, remain a reliable method, although further refinement could enhance their capability to consistently recognize more complex CAPTCHA patterns.

## 6. Discussion

This research presents ProCAPTCHA, a novel approach to enhancing online security, aiming to mitigate the challenges posed by sophisticated cyber threats, including automated bot attacks and human-powered CAPTCHA farms. This new era of authentication paradigm involves the integration of some personal skills and user-specific keystroke dynamics. Thus, more secure and highly individualized user authentication methods can be obtained. This discussion delves into the implications, effectiveness, and potential areas for improvement of the ProCAPTCHA system, as revealed through comprehensive performance evaluation and security analysis.

### 6.1 Implications of ProCAPTCHA implementation

#### 6.1.1 Enhanced security through personalization

ProCAPTCHA leverages the unique typing patterns of individuals, merging them with CAPTCHA challenges to create a dual-layered security mechanism. This personalization significantly elevates the difficulty for unauthorized entities to replicate or guess the CAPTCHA, as evidenced by the extremely low probabilities of successful breaches. The use of digraphs further complicates these challenges, requiring precise reproduction of not only the characters but also the specific timing between them, a feat nearly impossible for automated bots and CAPTCHA farms to achieve accurately.

#### 6.1.2 Balance between security and user experience

One of the standout features of ProCAPTCHA is its attempt to maintain a balance between enhanced security measures and user experience. By designing CAPTCHA challenges around familiar user data (such as names and phone numbers) and incorporating user-specific keystroke dynamics, ProCAPTCHA aims to ensure that legitimate users can navigate the authentication process with relative ease while still providing robust defense against unauthorized access.

### 6.2 Effectiveness against automated and human attacks

#### 6.2.1 Resilience against automated bots

To enhance the discussion on the effectiveness of ProCAPTCHA against automated and human attacks, specifically in this section, it’s crucial to delve deeper into the probability analysis provided in Section 5.1.1. This analysis underscores the formidable security posture of ProCAPTCHA by quantitatively demonstrating the minuscule chances of successful intrusion.

The probability of successfully breaking through ProCAPTCHA, as delineated in Section 5.1.1, is derived from the intricate combination of user-specific keystroke dynamics and the inclusion of digraphs within the CAPTCHA challenges. This analysis leveraged a mathematical model to calculate the likelihood of an attacker, whether human or bot, correctly guessing or solving a ProCAPTCHA challenge without prior knowledge of these unique behavioral biometrics. The results from this analysis revealed probabilities that were extraordinarily low, converging towards zero, effectively illustrating the system’s resilience against unauthorized access attempts.

For instance, the probabilities of an attacker correctly guessing the ProCAPTCHA challenges were calculated for various series, ranging from simple CAPTCHAs consisting of single characters to more complex ones incorporating multiple digraphs. These probabilities were found to be exceedingly low across all scenarios, with values such as 8.07×10^−12^ for a ProCAPTCHA challenge like "n12j14hn," which includes both single characters and digraphs. This incredibly low probability highlights the effectiveness of using personalized and dynamic CAPTCHA challenges in thwarting unauthorized access.

Furthermore, the digraphs add a layer of complexity that significantly lowers the probability of successful attacks. Digraphs require not only the correct identification of the characters but also the replication of the specific timing between them, a feature that is nearly impossible to accurately predict or replicate without intimate knowledge of the user’s typing behavior.

This quantitative analysis reinforces the conceptual underpinnings of ProCAPTCHA’s security mechanisms. The personalized nature of the challenges, combined with the statistical improbability of correct guesses, creates a robust barrier against potential intrusions. By utilizing a security approach that integrates both the complexity of the challenges and the unique behavioral patterns of the user, ProCAPTCHA effectively elevates the difficulty for unauthorized entities attempting to breach the system, whether through automated means or human-driven efforts.

The intricate composition of ProCAPTCHA, which deftly intertwines digraphs with unique keystroke dynamics and single characters, notably enhances its security posture, rendering it a robust mechanism against unauthorized breaches. This complexity is not merely superficial; it lies at the core of ProCAPTCH’’s design, making each challenge inherently unique and exceedingly difficult for intruders to predict or replicate. The inclusion of digraphs—pairs of characters typed in a specific sequence with precise timing—alongside the personalized rhythm of keystrokes for both single characters and digraphs elevates the authentication challenge to a level of personalized security. The probability analysis underpinning ProCAPTCHA’s efficacy reveals a near-zero likelihood of successfully breaching this security measure, highlighting the significant challenge intruders face in attempting to decipher these personalized and complex CAPTCHA challenges. This low probability of guessing, coupled with the bespoke nature of each ProCAPTCHA challenge, underscores the system’s effectiveness in deterring unauthorized access through a sophisticated blend of advanced security measures.

#### 6.2.2 Deterrence of human-powered CAPTCHA farms

ProCAPTCHA also addresses the challenge posed by CAPTCHA farms by integrating biometric data (keystroke dynamics) into the CAPTCHA challenge. This makes it exceedingly difficult for human solvers to bypass the system without access to the specific typing pattern of the legitimate user. This approach effectively neutralizes one of the most common third-party attacks on traditional CAPTCHA systems.

The evaluation of ProCAPTCHA using Gradient Boosting Trees, Decision Trees, and Random Forests highlighted the potential of machine learning algorithms in distinguishing between legitimate users and potential security threats. Gradient Boosting Trees, in particular, demonstrated consistently high performance across various complexity levels of ProCAPTCHA challenges, suggesting their suitability for this application. However, implementing such models in a real-world system would necessitate ongoing monitoring and updates to maintain their effectiveness against evolving attack methods. Furthermore, the computational demands of these models and the need for large datasets to train them effectively raise considerations regarding scalability and efficiency in deployment.

### 6.3 Potential areas for improvement

#### 6.3.1 Accessibility considerations

While ProCAPTCHA excels in security and personalization, it’s essential to consider the accessibility implications of relying heavily on keystroke dynamics. Users with physical impairments or those using alternative input methods may encounter difficulties with the system. Future iterations of ProCAPTCHA could explore adaptive mechanisms that can adjust to accommodate a wider range of user abilities and input methods, ensuring that security enhancements do not come at the expense of accessibility.

#### 6.3.2 Data privacy and security

The collection and use of biometric data (keystroke dynamics) for ProCAPTCHA generation necessitate stringent data privacy and security measures. Ensuring the confidentiality and integrity of this sensitive information is paramount. Adopting encrypted storage and secure transmission protocols, alongside transparent user consent processes, can mitigate potential privacy concerns and enhance user trust in the system.

### 6.4 Comparative analysis with other similar related works

The following table provides a comparative analysis of the proposed ProCAPTCHA method against several existing CAPTCHA methods, highlighting differences across various criteria.

According to Table 10, the comparative analysis of CAPTCHA methods from 2015 to 2024 reveals significant advancements and variations across different criteria. All methods in the study, including the proposed ProCAPTCHA, are user-friendly, indicating a consistent emphasis on usability. However, accessibility is specifically noted only for De Marsico et al. (2015) and ProCAPTCHA (2024), suggesting that many CAPTCHA methods could benefit from enhancements in this area. Starting from 2016, most CAPTCHA methods require specific hardware, reflecting a shift towards more sophisticated and secure solutions, though this might limit accessibility for some users. ProCAPTCHA does not require any specific hardware and can be used with just a mobile phone, making it exceptionally accessible and versatile.

**Table 10:** Differences between the proposed method and some existing methods.

Year	2003 [4]	2004 [14]	2008 [15]	2014 [21]	2015 [40]	2016 [16]	2016 [24]	2017 [28]	2017 [25]	2018 [20]	2018 [23]	2019 [17]	2020 [31]	Proposed method
CAPTCHA method	Von Ahn et al.	Rusu and Govindaraju	Chow et al.	Google’s "No CAPTCHA reCAPTCHA"	De Marsico et al. (FATCHA)	NuCAPTCHA	Hupperich et al. (SensorCAPTCHAs)	Siripitakchai et al. (EYE-CAPTCHA)	Pedometric	Frank et al.	Guerar et al. (CAPPCHA)	Kim S, Choi S (DotCHA)	Acien et al. (BeCAPTCHA-Mouse)	ProCAPTCHA
Attributes														
User-friendly	**✓**		**✓**	**✓**	**✓**	**✓**	**✓**	**✓**	**✓**		**✓**	**✓**	**✓**	**✓**
Accessibility				**✓**	**✓**									**✓**
Hardware requirement		**✓**				**✓**	**✓**	**✓**	**✓**	**✓**	**✓**	**✓**	**✓**	
Biometric integration								**✓**					**✓**	**✓**
Effective against bots	**✓**	**✓**	**✓**	**✓**	**✓**	**✓**	**✓**	**✓**	**✓**	**✓**	**✓**	**✓**	**✓**	**✓**
Effective against CAPTCHA farm				**✓**		**✓**	**✓**	**✓**	**✓**		**✓**	**✓**	**✓**	**✓**
Ease of implementation	**✓**			**✓**		**✓**	**✓**	**✓**	**✓**		**✓**	**✓**	**✓**	**✓**
Cost of implementation	**✓**			**✓**		**✓**	**✓**	**✓**	**✓**		**✓**	**✓**	**✓**	**✓**
User experience			**✓**	**✓**	**✓**	**✓**	**✓**	**✓**	**✓**		**✓**	**✓**	**✓**	**✓**
Effectiveness in Preventing Automated Script Attacks	**✓**	**✓**	**✓**	**✓**	**✓**	**✓**	**✓**	**✓**	**✓**	**✓**	**✓**	**✓**	**✓**	**✓**
Effectiveness in Preventing Phishing Attacks						**✓**								**✓**
Effectiveness in Preventing Brute Force Attacks						**✓**	**✓**	**✓**	**✓**		**✓**	**✓**	**✓**	**✓**

ProCAPTCHA also stands out for its integration of biometric data, specifically using unique keystroke dynamics of each user by incorporating single characters and digraphs from the user’s profile information. This approach is shared only with Siripitakchai et al. (2017) and demonstrates an evolution towards incorporating advanced security measures. All methods listed are effective against bots, demonstrating that this is a fundamental requirement for CAPTCHA solutions. From 2016 onwards, all methods, including ProCAPTCHA, are designed to be effective against CAPTCHA farms, indicating a focus on combating more sophisticated automated attacks.

Ease of implementation and cost-effectiveness are consistent features from 2016 onwards, ensuring these methods can be widely adopted and scaled across different platforms. User experience remains a priority, with all methods providing a good user experience, aligning with the user-friendly criterion, and ensuring that security measures do not compromise usability. Effectiveness in preventing automated script attacks is a common feature across all methods. However, only NuCAPTCHA (2016) and ProCAPTCHA (2024) are noted for their ability to prevent phishing attacks, highlighting ProCAPTCHA’s advanced security capabilities compared to earlier methods.

Starting in 2016, most methods, including ProCAPTCHA, have been effective against brute force attacks, indicating ongoing improvements in addressing this threat. In summary, ProCAPTCHA stands out for its comprehensive approach, integrating user-friendly design, accessibility, no hardware requirements, and the use of biometric data through single characters and digraphs from user profiles. This combination ensures robust security against various forms of attacks and reflects an evolution in CAPTCHA technology towards more inclusive and advanced security solutions.

## 7. Conclusion

ProCAPTCHA represents a significant advancement in CAPTCHA technology, effectively enhancing online security by integrating personalized keystroke dynamics with biometric data. This innovative approach not only distinguishes between human users and automated bots but also personalizes the authentication process, providing a robust defense against unauthorized access. By leveraging unique typing patterns derived from each user’s personal information—names, surnames, emails, and phone numbers—ProCAPTCHA extracts unique digraphs and single characters that are tailored to individual profiles, including specific times; those are dwell time, interval time, up-to-up time, and latency. These factors can enhance the security of each challenge.

The combination of single and digraph characters initiates the complication in the replication of keystroke patterns, making it exceedingly difficult for unauthorized parties to guess. The statistical analysis of keystroke dynamics confirms their effectiveness, as the results consistently return probabilities close to zero, indicating a highly secure and personalized authentication process. For instance, the likelihood of an attacker correctly guessing a ProCAPTCHA challenge like "n12j14hn," which includes both single characters and digraphs, is as low as 8.07×10^(-12), demonstrating the formidable security offered by ProCAPTCHA. Additionally, legitimate users successfully solve ProCAPTCHA challenges 98% of the time, ensuring a smooth user experience.

Moreover, incorporating advanced machine learning models, particularly Gradient Boosting Trees, plays a crucial role in enhancing ProCAPTCHA’s ability to accurately differentiate between legitimate users and potential threats across various CAPTCHA configurations. These models achieve an accuracy rate above 98% in distinguishing between legitimate users and automated threats, further strengthening the system’s security. This approach not only secures digital environments against automated threats but also effectively counters the prevalent issue of 3^rd^ party CAPTCHA farms, making a significant over traditional CAPTCHA methods. ProCAPTCHA is highly effective at stopping automated and human-powered CAPTCHA attacks. Its design makes it nearly impossible for unauthorized users or bots to break through. The chances of breaching ProCAPTCHA are extremely low, almost zero. This ensures strong security and reliable user verification.

This ProCAPTCHA approach not only bolsters security but also personalizes the user experience, ensuring that each CAPTCHA is uniquely challenging yet accessible to the intended user. This dual aspect of security and personalization sets a new standard in CAPTCHA technology, offering significant enhancements over traditional methods by effectively countering sophisticated threats while maintaining user convenience and trust.
